# In Vitro and In Silico Analysis of New n-Butyl and Isobutyl Quinoxaline-7-carboxylate 1,4-di-*N*-oxide Derivatives against *Trypanosoma cruzi* as Trypanothione Reductase Inhibitors

**DOI:** 10.3390/ijms232113315

**Published:** 2022-11-01

**Authors:** Alonzo González-González, Oscar Sánchez-Sánchez, R. Luise Krauth-Siegel, Maria Laura Bolognesi, Rogelio Gớmez-Escobedo, Benjamín Nogueda-Torres, Lenci K. Vázquez-Jiménez, Emma Saavedra, Rusely Encalada, José Carlos Espinoza-Hicks, Alma D. Paz-González, Gildardo Rivera

**Affiliations:** 1 Laboratorio de Biotecnología Farmacéutica, Centro de Biotecnología Genómica, Instituto Politécnico Nacional, Reynosa 88710, Mexico; 2 Center of Biochemistry, Heidelberg University, Im Neuenheimer Feld 328, 69120 Heidelberg, Germany; 3 Department of Pharmacy and Biotechnology, Alma Mater Studiorum, University of Bologna, I-40126 Bologna, Italy; 4 Departamento de Parasitología, Escuela Nacional de Ciencias Biológicas Instituto Politécnico Nacional, Ciudad de Mexico 07738, Mexico; 5 Departamento de Bioquímica, Instituto Nacional de Cardiología Ignacio Chávez, Ciudad de Mexico 14080, Mexico; 6 Facultad de Ciencias Químicas, Universidad Autónoma de Chihuahua, Chihuahua 31125, Mexico

**Keywords:** Chagas disease, trypanothione reductase, chemical synthesis, trypomastigotes, quinoxaline-1,4-di-*N*-oxide

## Abstract

American trypanosomiasis is a worldwide health problem that requires attention due to ineffective treatment options. We evaluated n-butyl and isobutyl quinoxaline-7-carboxylate 1,4-di-*N*-oxide derivatives against trypomastigotes of the *Trypanosoma cruzi* strains NINOA and INC-5. An in silico analysis of the interactions of 1,4-di-*N*-oxide on the active site of trypanothione reductase (TR) and an enzyme inhibition study was carried out. The n-butyl series compound identified as T-150 had the best trypanocidal activity against *T. cruzi* trypomastigotes, with a 13% TR inhibition at 44 μM. The derivative T-147 behaved as a mixed inhibitor with Ki and Ki’ inhibition constants of 11.4 and 60.8 µM, respectively. This finding is comparable to the TR inhibitor mepacrine (Ki = 19 µM).

## 1. Introduction

No efficient drug treatment has been developed in the more than 110 years since the discovery of the causal agent of American trypanosomiasis or Chagas disease. For this reason, it continues to be one of the most prevalent parasitic diseases, with 6 to 7 million people infected worldwide [1]. The increase in human migration from endemic countries and vector migration resulting from climate change have increased the incidence of this disease in non-endemic areas, making it a worldwide problem [2].

The variable efficacy of benznidazole (Bnz) and nifurtimox (Nfx) in the acute and chronic phases of *Trypanosoma cruzi* (*T. cruzi*) infection and their high human toxicity have resulted in treatment abandonment [3,4,5]. Therefore, more effective, and less toxic new treatments are necessary [6,7]. 

In the last decade, the search for new drugs has focused on pharmacological targets [8] such as *trans*-sialidase [9,10,11,12,13,14], cruzain [15,16,17,18], superoxide dismutase [19,20], triose phosphate isomerase [7,21], and trypanothione reductase (TR) [5,22,23,24]. TR plays a central role in the *T. cruzi* redox system serving as the enzyme responsible for trypanothione reduction after its oxidation, contributing to oxidative stress relief [25]. TR is essential and exclusive in trypanosomatids, so it is considered an attractive drug target [26,27].

Quinoxalines have a wide spectrum of biological activities [28] as anticancer [29,30,31], antimycobacterial [32,33], antibacterial [34,35], and antiparasitic [36,37,38,39,40] agents. Various quinoxaline-1,4-di-*N*-oxide derivatives have demonstrated trypanocidal activity. Ancizu et al., reported 3-cyano quinoxaline 1,4-di-*N*-oxide derivatives with a growth inhibition percentage (GI%) of *T. cruzi.* Most of these compounds had a low GI%, suggesting that modifications may improve their biological activity [41]. Later, Torres et al., reported a series of quinoxaline derivatives with ester groups at the 2-position with a half-maximal inhibitory concentration (IC_50_) in the low micromolar range [42]. This increase in activity with the addition of esters is related to an increase in solubility, suggesting the importance of the presence of esters in the quinoxaline ring. Methyl and ethyl esters of quinoxaline 1,4-di-*N*-oxide derivatives were synthesized and identified by Villalobos-Rocha as TR inhibitors (TRIs) by molecular docking studies [43]. Additionally, Chacon-Vargas et al., tested n-propyl and isopropyl substitutions at the 7-position to determine the trypanocidal and enzymatic activities of branched vs. unbranched aliphatic chains. Their results demonstrated that 7-isopropyl quinoxaline-carboxylate 1,4-di-*N*-oxide (T-085) derivatives cause TR inhibition through a non-competitive mechanism (Ki = 35 μM) [44]. This inhibitory effect proved to be non-selective, but the steric effect at the 7-position on the quinoxaline ring was necessary for inhibition. With this starting point, in this study, a new series of n-butyl quinoxaline-7-carboxylate 1,4-di-*N*-oxide derivatives were proposed for aliphatic chain elongation to increase liposolubility. Additionally, isobutyl ester derivatives were proposed to determine the isomer effect on biological activity against *T. cruzi* trypomastigotes. Finally, both series were evaluated in silico and in vitro against TR to confirm their mechanism of action. 

## 2. Results

### 2.1. Synthesis

**Figure 1:**
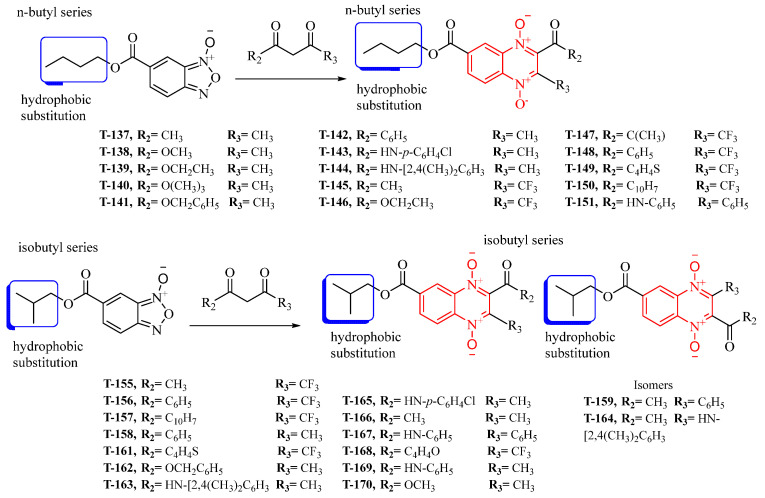
The synthetic pathway for forming the n-butyl and isobutyl quinoxaline 1,4-di-*N*-oxide series from the Beirut reaction.

Thirty new n-butyl and isobutyl quinoxaline-7-carboxylate 1,4-di-*N*-oxide derivatives were synthesized using the Beirut reaction with a yield ranging from 1.02 to 22.5% (Figure 1). All synthesized compounds were structurally elucidated using infrared (IR), proton, and carbon nuclear magnetic resonance (^1^H-NMR and ^13^C-NMR), and ultra-performance liquid chromatography-mass spectrometry (UPLC-MS) (Supplementary Material) before the biological evaluation.

The IR spectra showed characteristic bands for the n-butyl- and isobutyl quinoxaline-7-carboxylate 1,4-di-*N*-oxide derivatives at 1750–1660 (ν cm^−1^) corresponding to C=O and at 1300–1340 corresponding to an N-O bond. The ^1^H-NMR spectra confirmed the presence of n-butyl ester (triplet (~1 ppm), multiplet (~1.5–2 ppm), quartet (~3.6–3.8 ppm)) and isobutyl ester (doublet (~1.02–1.11 ppm), multiplet (~2.10–2.34 ppm), and doublet (~4.21–4.25 ppm)). UPLC-MS chromatograms showed single detection signals with measured masses equivalent to the molecular ion mass ±1, suggesting pure compound isolation. 

### 2.2. Biological Evaluation

#### Trypanocidal Activity against Trypomastigotes

Quinoxaline derivatives were initially evaluated against trypomastigote at a single fixed concentration of 50 µg/mL (Table 1). NINOA strain mortality ranged from 8.7 to 78.3%. For the reference drugs, Nfx and Bnz, it was 76.6% and 68.8%, respectively. Four compounds, T-142, T-168, T-169, and T-170, had a similar or higher trypanocidal activity than the reference drugs. 

The mortality percentages of the INC-5 strain were 11.80% to 74.8%. Ten compounds, T-140, T-146, T-147, T-150, T-155, T-163, T-167, T-168, T-169, and T-170 had a similar or higher trypanocidal activity than the reference drugs. Notably, T-167, T-169, and T-170, had a mortality percentage higher than 60%. 

The half-maximal lytic concentration (LC_50_) analysis showed that multiple compounds derived from these two series had better trypanocidal activity than the reference drugs Nfx and Bnz against the two strains tested. Compounds T-141, T-150, and T-169 showed the best activity of both series with LC_50_ values of 38.9 and 118, 64.3 and 81.5, and 56.9, and 62.2 µM for NINOA and INC-5, respectively, compared to 70.4 and 139.4, and 130.7 and 191.3 µM for Nfx and Bnz, respectively. These findings reinforce the importance of the presence of aromatic substituents in quinoxaline derivatives. T-141 featured a benzyl ester group, T-150, a naphthyl ketone, and T-169, a benzamide.

### 2.3. Molecular Docking on TcTR

Molecular docking analysis on the active site of *Tc*TR showed that seven (T-149, T-150, T-151, T-157, T-162. T-163, and T-167) of the thirty quinoxaline-7-carboxylate 1,4-di-*N*-oxide derivatives had a similar or lower predicted free energy of binding (FEB) than the natural ligand trypanothione disulfide (Table 2). Based on the structure of the substituents present in these quinoxalines, it may be suggested that the presence of large aromatic groups and protonable amines tends to increase the affinity toward the catalytic site of *Tc*TR. The interaction profile (Supplementary Table S1) and 2-D representation (Supplementary Table S2) for all evaluated ligands are included in the Supplementary Material.

**Figure 2:**
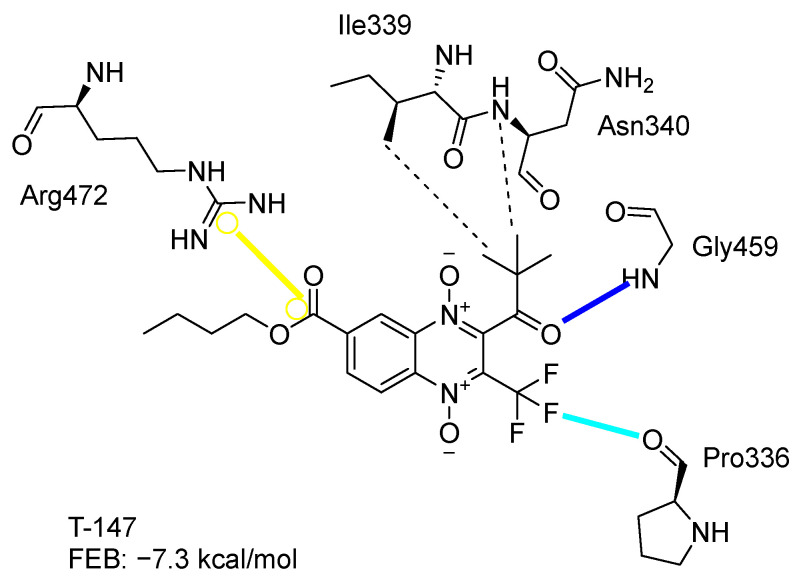
Two-dimensional interaction representation for T-147, a potential *Tc*TR inhibitor; dashed grey line: hydrophobic interaction; solid blue line: hydrogen bond; solid golden line: salt bridge; solid light cyan line: halogen interaction.

Figure 2 presents the interaction profile for T-147, which closely resembles the previously reported inhibitor T-085. A 3D representation for T-147 and trypanothione disulfide bound to *Tc*TR is included in the Supplementary Material.

### 2.4. Enzymatic Activity Evaluation

**Figure 3:**
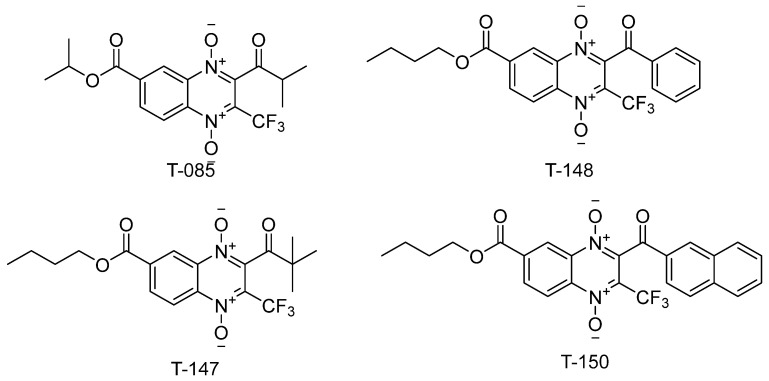
n-butyl quinoxaline-7-carboxylate 1,4-di-*N*-oxide derivatives tested for TR inhibitory activity and similarity to the known inhibitor, T-085.

Three (T-147, T-148, and T-150) n-butyl derivatives bearing a trifluoromethyl group were selected (Figure 3) to test their capacity to inhibit TR. These compounds showed a high mortality percentage and structural similarity to the known quinoxaline-derived TR inhibitor, T-085 [44].

#### Trypanothione Reductase Inhibition

The selected compounds were analyzed at two fixed inhibitor concentrations, 20 and 5 µM, and two substrate concentrations of 100 and 44 µM TS_2_. At 20 μM, T-147 and T-148 inhibited 35 and 36%, respectively, but T-150 was insoluble. At 5 μM, T-147 and T-148 yielded 18% inhibition, whereas T-150 showed 13% inhibition (Table 3).

**Figure 4:**
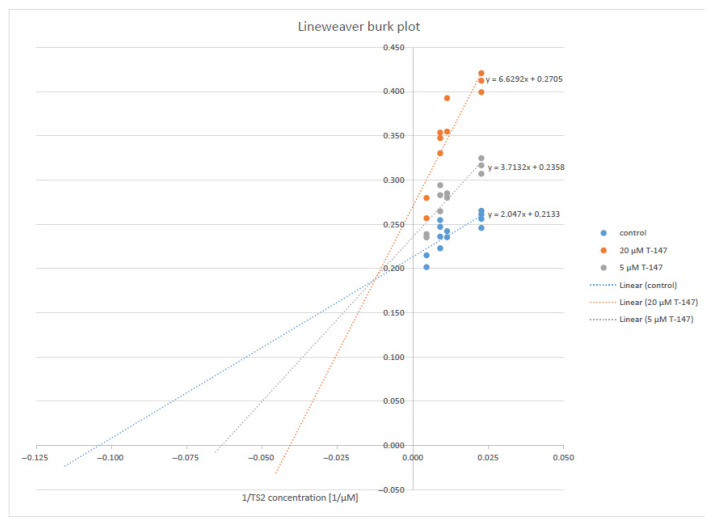
Lineweaver–Burk plot of TR inhibition by the compound T-147.

Compound T-147 (Figure 4) was studied at two fixed inhibitor concentrations (5 and 20 μM) to evaluate the type of inhibition in more detail, varying the trypanothione disulfide concentrations (22, 44, 88, 110, and 220 µM) and saturating NADPH. A Lineweaver–Burk plot revealed a mixed-type inhibition with respect to trypanothione disulfide. The inhibitor constants, Ki and Ki’ of 11.4 and 60.8 µM, respectively, were calculated by non-linear regression using Graph Pad Prism software (version 5).

### 2.5. Molecular Dynamics on TcTR

A molecular dynamics analysis was performed for the compound that behaved as the TR inhibitor, T-147, and the known TR inhibitor, mepacrine. The dynamics were analyzed with three measurements: root-mean-square differentiation (RMSD), root-mean-square fluctuation (RMSF), and radius of gyration.

#### 2.5.1. RMSD Analysis

**Figure 5:**
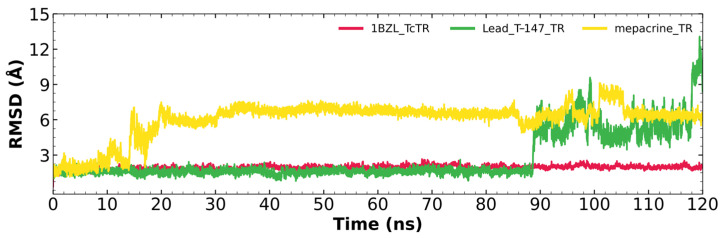
RMSD graph for fluctuations over time for *Tc*TR complexes and free *Tc*TR; fluctuations: T-147 (0.79–12.28 Å), mepacrine (1.02–8.10 Å), and *Tc*TR (0.33–2.22 Å).

Figure 5 shows the RMSD fluctuations for the T-147-*Tc*TR complex (green), mepacrine-*Tc*TR complex (yellow), and free *Tc*TR (red). The mepacrine-TcTR complex shows a maximum fluctuation of 8.1 Å and maintains a fluctuation in the range of 3–6 Å with a mean of 6.00 ± 1.53 Å. While, the T-147-*Tc*TR complex shows a maximum fluctuation of 12.28 Å, which can be observed at the end of the dynamics; still, the dynamics remain stable for the first 88 ns with a fluctuation of around 2 Å. The overall fluctuation had a mean of 2.68 ± 2.01 Å.

#### 2.5.2. RMSF Analysis

**Figure 6:**
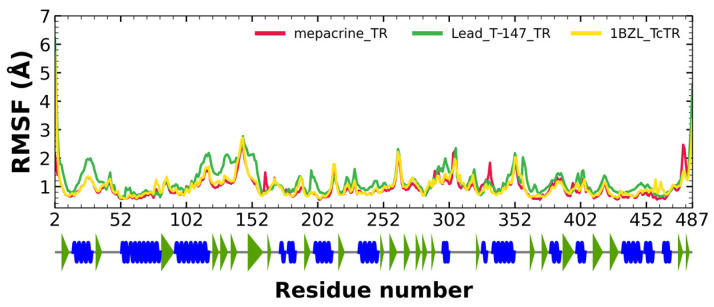
RMSF graph for fluctuations over time for mepacrine-*Tc*TR and T-147-*Tc*TR complexes, and apo-*Tc*TR, blue spiral (alpha helix), green triangle (beta sheet), in between space (loop).

RMSF fluctuations for the complexes, T-147-*Tc*TR (green), mepacrine-*Tc*TR (red), and free *Tc*TR (yellow), are shown in Figure 6. The fluctuations have only minor differences with respect to the apoprotein, suggesting stable binding without affecting residues outside the active site.

#### 2.5.3. Radius of Gyration

**Figure 7:**
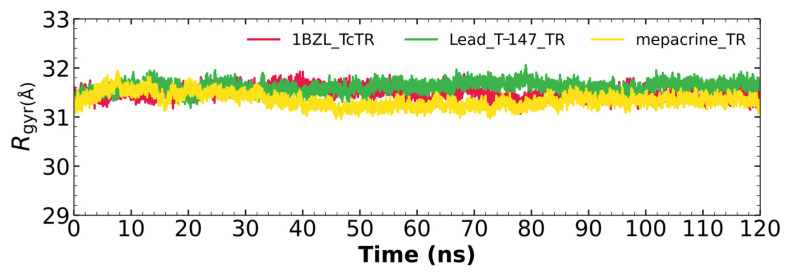
The radius of gyration graph for molecular dynamics over time for mepacrine-*Tc*TR (yellow) and T-147-*Tc*TR (green) complexes, and apo-*Tc*TR (red).

Figure 7 shows the radius of gyration for the mepacrine-*Tc*TR and T-147-*Tc*TR complexes, and apo-*Tc*TR. This graph shows a mean radius of gyration between 31 and 32 Å.

### 2.6. In Vitro Selectivity Assessment of Enzymatic Activity

The effect of T-147 on human glutathione reductase (hGR) was determined to assess the selectivity of compound T-147 towards the parasite TR. The degree of inhibition of human GR was measured at two fixed concentrations (5 µM and 20 µM) of T-147 in the presence of 37 or 92 µM glutathione disulfide (GSSG) saturating NADPH. The results are shown in Table 4.

A second experiment was conducted to determine the type of inhibition and the inhibition constant at two fixed concentrations of the inhibitor (5 and 20 μM), varying the glutathione disulfide concentrations (21.5, 43, 86, 107.5, 372, and 930 µM) and saturating NADPH. The compound was a non-competitive inhibitor of hGR with a Ki value of 25 µM. The kinetic constant was obtained by non-linear regression of the experimental data using Graph Pad Prism (version 5). In addition, a Lineweaver–Burk plot that directly visualized the non-competitive type of inhibition was prepared (Supplementary Figure S1).

### 2.7. Molecular Dynamics on hGR

To further analyze selectivity, a molecular dynamics analysis was performed with hGR for the compound found to behave as a TR inhibitor, T-147, and for the known TR inhibitor, mepacrine. The dynamics were analyzed with three measurements: RMSD, RMSF, and radius of gyration.

#### 2.7.1. RMSD Analysis

**Figure 8:**
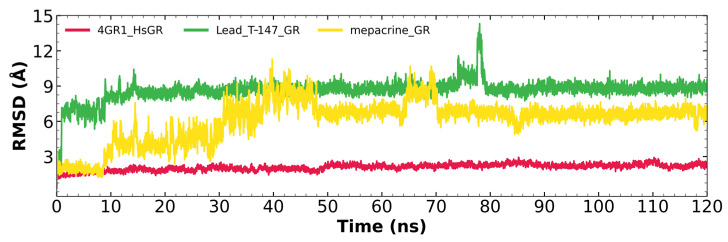
RMSD graph for fluctuations over time for hGR complexes; fluctuation: T-147 (0.81–14.31 Å), mepacrine (0.91–11.3 Å), and hGR (0.32–2.93 Å).

Figure 8 shows the RMSD fluctuations for the T-147-hGR (green) and mepacrine-hGR (yellow) complexes, and free hGR (red). The mepacrine-hGR complex showed a maximum fluctuation of 11.3 Å and fluctuations in the range of 4–7 Å with a mean of 6.11 ± 1.75 Å. While, the T-147-hGR complex showed a maximum fluctuation of 14.31 Å, which can be observed at 78 ns. Most of the dynamics remained stable at about 9 Å. The overall fluctuation had a mean of 8.62 ± 0.88 Å.

#### 2.7.2. RMSF Analysis

**Figure 9:**
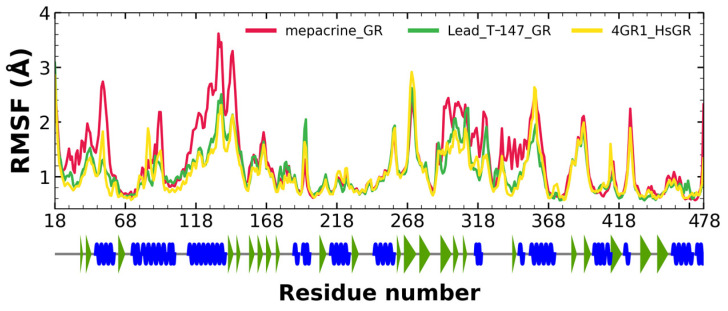
RMSF graph for fluctuations over time for mepacrine-hGR, T-147-hGR complexes, and apo-hGR, blue spiral (alpha helix), green triangle (beta sheet), in between space (loop).

RMSF fluctuations for the complexes, T-147-hGR (green) and mepacrine-*Tc*TR (red), and free hGR (yellow) are shown in Figure 9. The fluctuations had minor differences with respect to the apoprotein in the residue with ranges of 118–158, 288–298, and 328–348, suggesting unstable binding since it minorly affects residues outside the intended binding site.

#### 2.7.3. Radius of Gyration

**Figure 10:**
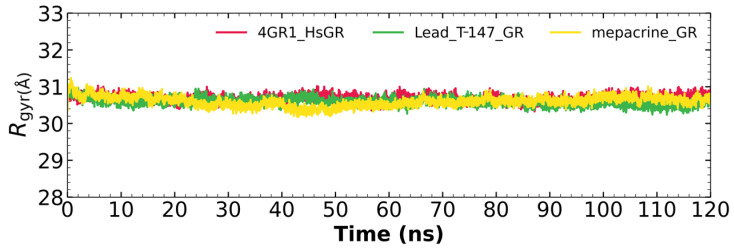
The radius of gyration graph for molecular dynamics over time for mepacrine-hGR (yellow) and T-147-hGR complexes (green), and apo-hGR (red).

Figure 10 shows the radius of gyration for the mepacrine-hGR and T-147-hGR complexes, and apo-hGR. This graph shows a mean radius of gyration between 30.5 and 31.5 Å.

## 3. Discussion

### 3.1. Biological Activity

#### 3.1.1. Trypanocidal Activity

The LC_50_ values obtained for the n-butyl series against the NINOA strain were 38.9 to 167 µM. Nine out of the fifteen compounds were under 100 µM. Compared to the reference drugs, Bnz (130.7 µM) and Nfx (70.4 µM), they had comparable or lower LC_50_ values. In the case of the INC-5 strain, LC_50_ values for n-butyl derivatives were in the range of 81.5 to 269.5 µM. Eight out of the fifteen compounds were below 130 µM. Compared to the reference drugs, Bnz (191.3 µM) and Nfx (139.4 µM), they had lower LC_50_ values.

Regarding the isobutyl series against the NINOA strain, LC_50_ values were 33.3 to 556 µM. Four of the fifteen compounds were under 100 µM, comparable to or lower than the reference drugs. The LC_50_ values obtained for the isobutyl series against the INC5 strain were 62.2 to 233 µM. Nine of the fifteen compounds were below 136 µM, lower than the reference drugs. The LC_50_ ranges found herein were comparable to or lower than the reference drugs. 

Reports of the half-maximal cytotoxic concentration values (CC_50_) for quinoxaline derivatives suggest that multiple derivatives from both series may act as trypanocidal agents at concentrations safe for mammalian cells. Quinoxaline-1,4-di-*N*-oxide derivatives reported by Chacon-Vargas et al., 2017, and analogous to those reported here, showed mean cytotoxic concentrations (CC_50_) against macrophages in the range of 6.7–325.61 µM on equivalence with reference drugs Nfx and Bnz with CC_50_ 201.05 and 352.01 µM, respectively. These derivatives had LC_50_ values of 2.42–238.28 µM with a desired higher trypanocidal effect rather than a cytotoxic effect for multiple derivatives [44]. Perez-Silanes et al., 2016 reported quinoxaline derivatives with IC_50_ values of 0.6 to 12.1 µM for the highest trypanocidal compounds compared to CC_50_ values for VERO cells of 3–454.7 µM, with all these compounds having higher antiparasitic than cytotoxic activity [45]. Quinoxaline derivatives reported by Estevez et al. showed a trypanocidal effect with an IC_50_ of 11.5 to >25 µM and cytotoxic activity against VERO cells of 7.6 to >254 µM [36]. Taken together, these studies suggest that quinoxalines can potentially be used safely against parasites as they have lower cytotoxicity than trypanocidal activity.

#### 3.1.2. Structure-Activity Relationship

The n-butyl derivative with the highest mortality percentage against trypomastigote form against both strains of the parasite was T-150, and for isobutyl derivatives, compounds T-167 and 169. On the one hand, T-150 has a naphthyl group at the 2-position and a trifluoromethyl group at the 3-position. In contrast, T-167 and T-169 bear a benzamide substituent at the 2-position, and phenyl and methyl at 3-position respectively. These results support the importance of bulky, aromatic, and halogenated substituents to favor biological activity. In contrast to these results, the analogs of these compounds with methyl and ethyl esters at the 7-position in the study by Villalobos-Rocha et al. [43] and isopropyl in the study by Chacon-Vargas et al. [39] did not show outstanding results suggesting that perhaps a longer chain for the ester at the 7-position allows enhanced activity.

Derivatives with bulky aromatic or trifluoromethyl groups were among the most active compounds against trypomastigotes. A similar comparison revealed that some compounds benefited from the 7-position substitution of n-butyl compared to isobutyl (T-137 vs. T-166, both strains; T-141 vs. T-162, both strains; T-142 vs. T-158 and T-159, both strains; and T-148 vs. T-156 and T-149 vs. T-161, both strains). Two compounds showed lower LC_50_ toward INC-5 when substituted with isobutyl (T-163, and T-155 vs. T-144 and T-145). Derivatives without trifluoromethyl substituents (T-137 vs. T-145, T-139, and T-146; T-142 vs. T-148; and T-167 vs. T-155) were favored against NINOA strain trypomastigotes. Compared to the presence of alkyl vs. alkoxide, a slight increase was observed in the activity of ester-containing compounds compared to ketone-containing compounds for the NINOA stain. Aliphatic esters at the 2-position, in a trypomastigote assay of NINOA strain, had similar activity, with tert-butyl substituted T-140 being the lowest compared to aromatic benzyl ester T-141 which has the highest activity among all the esters. This tendency is different in the INC-5 strain, where the tert-butyl-substituted T-140 was the most potent ester, and the aromatic benzyl ester T-141 had slightly less activity. Compounds T-143 and T-144, which have benzamides at the 2-position, had activities higher than 50% for the NINOA strain. These compounds had a notable trypanocidal activity against this parasite form. In contrast, their isobutyl analogs did not show an activity higher than 30% against NINOA, yet T-163 displayed an activity higher than 50% against the INC-5 strain. 

### 3.2. Molecular Docking on Trypansoma cruzi Trypanothione Reductase (TcTR)

The interaction predicted by molecular docking with the PLIP software showed that the n-butyl and isobutyl series interact with previously reported *Tc*TR residues important for binding the natural ligand trypanothione disulfide (see PLIP interaction profile in Table 2, Supplementary Tables S1 and S2).

The most recurring predicted interactions for n-butyl-quinoxaline-7-carboxylate 1,4-di-*N*-oxide derivatives were with active site residues, Val-59, Ile107, Ile339, Phe-396, Leu-399, His-461, and Glu-466. Similarly, isobutyl quinoxaline-7-carboxylate 1,4-di-*N*-oxide derivatives had recurring predicted interactions with Ile-339, Asn-340, Phe-369, His-461, and Glu-466. The residues Leu-399 and Phe-396 are part of the denominated Z-site (subsite within the interphase catalytic site) reported to participate in the binding of the hydrophobic part of chlorpromazine, a known TR inhibitor; likewise, the interactions with Glu-466 and His-461 are considered important to the binding of the natural ligand [46,47]. According to the interaction profile of trypanothione disulfide, it can be predicted that both n-butyl and isobutyl quinoxaline derivatives may behave as TR inhibitors. Figure 2 shows the interaction profile predicted for the new quinoxaline-1,4-di-*N*-oxide derivative that behaves as a TR inhibitor. No predicted interactions are shared with the natural ligand, which is congruent with the mixed-inhibitory activity found.

### 3.3. Trypanothione Reductase Inhibition

In accordance with previous reports [46,47], we suggest that n-butyl-quinoxaline-7-carboxylate-1,4-di-*N*-oxide derivatives may inhibit TR and, through this activity, contribute to the molecular action mechanism of trypanocidal activity observed against *T. cruzi*. As such, three (T-148, T-147, and T-150) derivatives bearing a trifluoromethyl group (Figure 5) were selected to test their capacity to inhibit TR. These compounds showed a high mortality percentage and structural similarity to the known quinoxaline-derived TR inhibitor, T-085 [44].

Firstly, the compounds were analyzed at a single sub-saturating substrate concentration of 44 µM TS_2_ (Km TS_2_ = 23 μM) [48]. At 20 μM (the highest non-precipitating concentration), T-148 showed an inhibition of 36%, followed by T-147 with a percentage inhibition of 35%; T-150 was insoluble (Table 3). Each compound was then evaluated at a concentration of 5 μM. Under these conditions, T-147 and T-148 yielded 18% inhibition, whereas T-150 lowered the activity by 13% (Table 3). In the presence of 100 µM TS_2_, T-148 and T-147 displayed a degree of inhibition similar to that seen at 44 µM TS_2_, suggesting that the inhibition may not be purely competitive. Though all compounds chosen for the enzymatic inhibition assay bear hydrophobic substituents, it is noteworthy that the structures of the two compounds with the most inhibition, T-147, and T-148, had the least bulky substituents, suggesting that they fit better at the inhibition site than the bulkier T-150. At the same time, T-150 showed a slight inhibition at a 44 µM concentration of TS_2_ but no inhibition at 100 µM TS_2,_ suggesting that if T-150 were to behave as an inhibitor, it would have a more competitive nature than T-147 and T-148. Even though T-148 and T-150 share an aromatic substituent at the 2-position, their behaviors are considerably different, which is probably related to the bulkiness of the latter.

### 3.4. In Silico Analysis of Inhibitor Binding TcTR

RMSD results (Figure 5) for mepacrine are consistent with the information that this compound is a confirmed trypanothione reductase inhibitor with a stable trajectory at about 6 Å. The behavior observed for T-147 is consistent with its inhibitory activity, maintaining an RMSD around 2 Å, close to the binding site of the natural ligand and bearing interactions with residues at the hydrophobic clefts, the Z-site and γ-Glu site. Analysis of RMSF graphs (Figure 6) for TR show minor fluctuations located at loop regions prone to fluctuations; still, proteins remain mostly stable through the dynamics suggesting that a ligand interaction did not considerably affect the protein. The most notable change in RMSF may be seen in the region 102–152, where the RMSF increased slightly for T-147 with respect to the apoprotein. This RMSF supports the notion that this complex formed with T-147 is stable at its binding site and does not considerably alter other regions. It is noteworthy in the graph of the radius of gyration (Figure 7) that complexes have a stable radius throughout the 120 ns of molecular dynamics. When comparing receptors to complexes, there is no major difference. This finding suggests that protein remains compact during its dynamics analysis. Altogether it is possible to consider this in silico prediction to be a close representation of the inhibitory activity of these compounds on *Tc*TR.

### 3.5. Glutathione Reductase Inhibition

The results obtained for the inhibitory assay of T-147 on human glutathione reductase show that the substrate concentration is not very relevant to the enzyme inhibition. This finding suggests that the compound may not directly interfere with disulfide binding.

Thus, both enzymes showed comparable Ki values for T-147, indicating that this derivative is not a selective TR inhibitor. Still, this result, together with the reported T-085 inhibitor [42], provides the basis for new chemical modifications to obtain a TR inhibitor with higher selectivity.

Modifications to R7 (from isopropyl to n-butyl) and R2 (from isopropyl to *tert*-butyl) on the quinoxaline ring of T-085 and T-147 modified the type of inhibition they had on TR. Considering this information, it is worthwhile to further investigate modifications to these positions to potentially attain a modification of the type of inhibition and selective inhibition of TR.

### 3.6. In Silico Analysis of Inhibitor Binding hGR

Additional molecular dynamics analysis permitted the finding that the stability of T-147 on hGR is significantly lower than on *Tc*TR since it has a mean RMSD of 8.62 Å (Figure 8) in contrast to 2 Å with *Tc*TR (Figure 5), suggesting better inhibition. These results support that T-147 may not directly interfere with disulfide binding since it moves considerably from the binding site. The results observed for RMSF (Figure 9) further emphasize the idea that T-147 moves from the initial binding site since there are fluctuations on at least three regions, residues in the ranges 118–158, 288–298, and 328–348; whereas there are nearly no fluctuation differences for *Tc*TR. Just as for *Tc*TR, the radius of gyration for hGR (Figure 10) remains constant for each complex and apoprotein, supporting the idea that the ligand does not mainly destabilize protein, thus remaining compact throughout the dynamics analysis.

## 4. Materials and Methods

### 4.1. Chemical Synthesis

All reagents were purchased from chemical vendor Sigma-Aldrich (Mexico). The n-butyl and isobutyl quinoxaline-7-carboxylate1,4-di-*N*-oxide derivatives (from T-137 to T-170) were synthesized using the Beirut reaction described by Gomez-Caro et al [49]. The corresponding β-diketone (10.6 mmol) was added to the solution of the appropriate benzofuroxan-*N*-oxide (2.4 mmol) in dry chloroform (35 mL) while on an ice bath. Triethylamine (TEA) was added (1 mL), and the reaction mixture was stirred at room temperature for 4–7 days. The infrared (IR) spectra were obtained using the PLATINUM-ATR Bruker Alpha FT-IR spectrometer. The ^1^H-NMR spectra were obtained in DMSO-d_6_ with trimethyl silane (TMS) as the internal standard using a 400 MHz Bruker Advance III spectrometer (AXS Inc., Madison, WI, USA). Ultra-high performance liquid chromatography analysis was carried out in a UPLC with ACQUITY QDa detector (ACQUITY H UPLC^®^ CLASS, Waters, Milford, MA, USA) using a 2.1 × 100 mm (ACQUITY UPLC^®^ BEH C_18_, 130 Å, 1.7 μm, WATERS S.A. de C.V. Ciudad de Mexico, Mexico) column with running conditions of 1 μL sample injection, formic acid 0.1% (*v*/*v* water) acetonitrile (30:70) mobile phase, 5 min per run and pressure from 0–15,000 psi. The purity and reaction progress were monitored with thin-layer chromatography (TLC) on silica gel 60-coated plates (PF-245, Merck; Tokyo, Japan) of 0.25 nm thickness. The resulting plates were observed under UV light at 254–265 nm. 

### 4.2. Biological Evaluation

#### 4.2.1. Parasite Culture

The *T. cruzi* NINOA (MHOM/MX/1994/NINOA) and INC-5 (MHOM/MX/97/Inc-5) strains were used for this study. These strains were isolated in Mexico from humans [50]. Epimastigotes were cultured in vitro in brain-heart infusion (BHI) supplemented with 10% fetal bovine serum (FBS) and 1% penicillin/streptomycin (500 µg/mL (Gibco, Thermo Fisher, Ciudad de Mexico, Mexico) at 28 °C. The cultures were kept at exponential growth. They were transferred to fresh media every 7 days. CD1 mice, 6 to 8 weeks old, were infected with *T. cruzi* trypomastigotes to obtain trypomastigote samples in blood. The experiments were conducted in accordance with NOM-062-Z00-1999, published on 22 August 2009.

#### 4.2.2. Trypanocidal Activity against Trypomastigotes

CD1 female mice, 6 to 8 weeks old, were infected with *T. cruzi* bloodstream trypomastigotes. The course of infection was followed for 4 to 6 weeks. At the peak of parasitemia (tracked every few days under a microscope), blood was drawn by cardiac puncture, and the sample was supplied with sodium heparin as an anticoagulant. The blood sample was adjusted to 1 × 10^6^ bloodstream trypomastigotes/mL.

In a 96-well microplate, 195 µL of infected blood was added to each well along with 5 µL of a solution at different testing concentrations of n-butyl and isobutyl quinoxaline-7-carboxylate 1,4-di-*N*-oxide, and the reference drugs to reach a final volume of 200 µL. Each concentration (3.25–50 μg/mL) was assayed in triplicate. Initially, all the compounds were tested at a final concentration of 50 μg/mL (103–157 µM). Wells with untreated trypomastigotes, DMSO 5%, served as a negative control. Wells with reference drugs were used as positive controls. The microplates were refrigerated at 4 °C for 24 h. The bloodstream trypomastigotes were quantified by the Brenner–Pizzi method [51]; 5 µL were placed on the microscope slide and covered with a 13 × 13 mm glass cover. The motile parasites were counted in 15 microscope fields at 40× with an optical microscope. The percentage of mortality was calculated by comparing untreated wells with treated wells. The half-maximal lethal concentration (LC_50_) was determined by Probit analysis.

### 4.3. Molecular Docking Analysis

Molecular docking analysis was conducted to evaluate the possible interaction of the n-butyl and isobutyl quinoxaline-7-carboxylate 1,4-di-*N*-oxide derivatives against *T. cruzi* TR. The ligand structures were drawn with Marvin Sketch 18.10, energy minimized with Chimera 1.12, and saved in pdb format. The protein receptor trypanothione reductase from *Trypanosoma cruzi Tc*TR structure was obtained from the Protein Data Bank (PDB), access code 1BZL (accessed August 2018). Water molecules, co-crystalized ligands, and ions were removed from the protein structure. It was then energy-minimized using the Yasara Minimization Server (accessed August 2018) before docking simulation. All docking simulations were performed with AutoDock Tools 4.2 [52].

The molecular docking analysis was done on the interphase catalytic site coordinate space (X = 65.805, Y = 5.055, and Z = 0.955) using 20 Å in each axis with 1 Å spacing in the Grid Box. The results were analyzed considering the highest estimated free energy of binding (FEB) for each protein-ligand complex, which was later analyzed to determine the molecular interactions between protein and ligand utilizing the Protein-Ligand Interaction Profiler (PLIP) version 2.2.2 [53].

### 4.4. Molecular Dynamics

Molecular dynamics analysis was performed with GROMACS version 2018.4 [54]. First, the topologies for T-147 and mepacrine were generated with ACPYPE with the general amber force field (GAFF). Solvation was done with water molecules in a dodecahedron with a minimum distance from the wall of 10 Å, using the TIP3P water model. The necessary ions (Na^+^ and Cl^−^) were added to have a neutral charge in the system. The system was energy-minimized by the steepest descent algorithm. Then, two equilibrium steps were performed. First, the compound was simulated at NVT conditions (constant number of particles, volume, and temperature). For the second step, the compound was simulated at NPT conditions (constant number of particles, pressure, and temperature). Each stage achieved a duration of 100 ps. Finally, the simulation was performed at a temperature of 300 K for a trajectory of 120 ns [55,56]. The stability of the complex was determined using GROMACS built-in tools. The RMSD for the α carbons and the ligand were obtained. The RMSD matrix was calculated for the 120 ns. The RMSF for the α carbons was done to understand the effect of the compound on the secondary structure of *Tc*TR and *Hs*GR. Finally, the radius of gyration of each complex was obtained to determine the stability of the complex and the tridimensional compactness of *Tc*TR and *Hs*GR.

### 4.5. Enzymatic Activity Evaluation

#### 4.5.1. Trypanothione Reductase

Natural ligand trypanothione disulfide (TS_2_) and recombinant *Trypanosoma brucei* TR were prepared following a previously reported methodology [57]. Stock solutions of inhibitors at a concentration of 5 mM were prepared in DMSO. Recombinant human glutathione reductase (hGR) was provided by Dr. Heiner Schirmer, Heidelberg, Germany. The kinetic analyses were conducted using a Jasco V 650 spectrophotometer and optical polystyrene cuvettes (10 × 4 × 45 mm). TR activity was determined at 25 °C in a total volume of 1 mL of TR assay buffer (40 mM HEPES, 1 mM EDTA, pH 7.5 containing 100 μM NADPH) and 5–10 mU of enzyme in the presence and absence of inhibitors [57,58]. Each assay contained, at most, a total of 5% DMSO, which is reported to be innocuous for enzymatic activity. The reaction was initiated by adding various concentrations of TS_2,_ and the consumption of NADPH at 340 nm was monitored. The activity was determined for TR inhibition in the absence and presence of two or three constant concentrations of the inhibitor at varying TS_2_ concentrations (20, 40, 60, 100, and 200 μM). The type of inhibition was evaluated from double reciprocal Lineweaver–Burk plots. The inhibition constants Ki were calculated by non-linear regression using Graph Pad Prism software (version 5) [42,59,60].

#### 4.5.2. Glutathione Reductase

GR activity was determined at 25 °C in a total volume of 1 mL of GR buffer assay (20.5 mM KH_2_PO_4_, 26.5 mM K_2_HPO_4_, 200 mM KCl, and 1 mM EDTA, pH 6.9). The assays contained 100 µM of NADPH, 5–10 mU of GR, and varying inhibitor concentrations. Each assay contained, at most, a total of 5% DMSO. The reaction was initiated by adding glutathione disulfide (GSSG) (37 and 92 µM), and the decrease of absorption at 340 nm was monitored. The activity of GR inhibition was determined in the absence and presence of two or three constant concentrations of the inhibitor at varying GSSH concentrations (21.5, 43, 86, 107.5, 372, and 930 µM). The type of inhibition was evaluated from double reciprocal Lineweaver–Burk plots. The inhibition constants Ki were calculated by non-linear regression using Graph Pad Prism software (version 5) [42,59,60].

## 5. Conclusions

In this study, novel n-butyl and isobutyl quinoxaline-7-carboxylate 1,4-di-*N*-oxide derivates were obtained. Compounds T-141, T-142, and T-170 had the lowest LC_50_ values against trypomastigotes of the NINOA strain. Compounds T-150, T-163, and T-169 had LC_50_ values against INC-5 strain lower than the reference drugs Bnz and Nfx. SAR analysis showed that aromatic substituents are predominant as a substitution at the 2-position to enhance biological activity. Interestingly, compound T-147 showed good trypanocidal activity against both parasite forms.

In silico analysis identified the importance of the presence of aromatic groups as substituents for potential TR inhibition. Eleven of the thirty compounds analyzed had a FEB lower than −8 kcal/mol (−8.1 to −9.9 kcal/mol). Ten of these eleven compounds had an aromatic ring as part of their structures, supporting the importance of this structure. The top derivative was T-150 with FEB −9.9 kcal/mol. Most interactions were seen with Z-site (Phe396, Pro398, and Leu399) and γ-Glu site (His461, Glu466, and Glu467), which are both subsites within the interphase catalytic site, portraying them as potential inhibitors. 

The enzymatic analysis of T-148, T-147, and T-150 against TR revealed that T-147 behaves as a mixed-type inhibitor with inhibition constants Ki and Ki’ values of 11.4 and 60.8 µM, respectively, comparable to the known TR inhibitor mepacrine (Ki = 19 µM) [48]. However, T-147 showed a non-competitive inhibition of hGR with a Ki of 25 µM, which renders the compound rather non-selective towards TR. These results warrant further research on new quinoxaline 1,4-di-*N*-oxide derivatives with trypanocidal activity that may act as specific TR inhibitors.

## Figures and Tables

**Table 1:** Trypanocidal activity of n-butyl-and isobutyl quinoxaline-7-carboxylate 1,4-di-*N*-oxide derivatives against *T. cruzi* trypomastigotes of the NINOA and INC-5 strains.

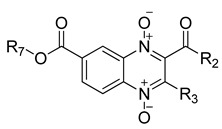							
Code	R_2_	R_3_	R_7_	Trypomastigotes			
				% mortality at 50 µg/mL		LC_50_ (µM)	
				NINOA	INC-5	NINOA	INC-5
T-137	-CH_3_	-CH_3_	CH_3_(CH_2_)_3_-	49.5 ± 2.1	27.2 ± 4.1	118.2 ± 2.2	192.6 ± 7.5
T-138	-OCH_3_	-CH_3_	CH_3_(CH_2_)_3_-	58.1 ± 1.3	40 ± 3.4	87.96 ± 3.6	269.5 ± 10
T-139	-OCH_2_CH_3_	-CH_3_	CH_3_(CH_2_)_3_-	58.2 ± 0.9	33.1 ± 1.9	96 ± 1.4	163 ± 5.2
T-140	-OC(CH_3_)_3_	-CH_3_	CH_3_(CH_2_)_3_-	45.3 ± 1.7	50.8 ± 4.6	122 ± 2.1	104 ± 5.6
T-141	-OCH_2_ C_6_H_5_	-CH_3_	CH_3_(CH_2_)_3_-	71 ± 1.9	40.2 ± 3.1	38.9 ± 1.4	118 ± 6.3
T-142	-C_6_H_5_	-CH_3_	CH_3_(CH_2_)_3_-	67.6 ± 3.5	36.2 ± 4.3	44.0 ± 2.6	160 ± 6.2
T-143	-HN-*p*-C_6_H_4_Cl	-CH_3_	CH_3_(CH_2_)_3_-	63.8 ± 3.2	37.8 ± 2.3	51.1 ± 1.9	121 ± 6.3
T-144	-NH-[2,4(CH_3_)_2_C_6_H_3_]	-CH_3_	CH_3_(CH_2_)_3_-	57.1 ± 1.8	37.4 ± 1.1	66.3 ± 4.5	122 ± 5.7
T-145	-CH_3_	-CF_3_	CH_3_(CH_2_)_3_-	28.6 ± 5.6	45 ± 3.1	167 ± 7.8	242 ± 7
T-146	-OCH_2_CH_3_	-CF_3_	CH_3_(CH_2_)_3_-	38.1 ± 1.9	48 ± 1.7	123 ± 5.2	222 ± 8
T-147	-C(CH_3_)_3_	-CF_3_	CH_3_(CH_2_)_3_-	45.2 ± 2.2	48.8 ± 2.4	116 ± 5.1	124 ± 3.6
T-148	-C_6_H_5_	-CF_3_	CH_3_(CH_2_)_3_-	56.7 ± 3.7	35 ± 1.6	65.3 ± 2.1	128 ± 4.4
T-149	-C_4_H_3_S	-CF_3_	CH_3_(CH_2_)_3_-	48.1 ± 1.6	34.3 ± 2.7	96.1 ± 4.1	135 ± 3.8
T-150	-C_10_H_7_	-CF_3_	CH_3_(CH_2_)_3_-	53.3 ± 4.1	50.4 ± 1.9	64.3 ± 1.7	81.5 ± 1.4
T-151	-NH-C_6_H_5_	-C_6_H_5_	CH_3_(CH_2_)_3_-	41 ± 0.8	30.5 ± 1.8	130 ± 5.0	103 ± 4.2
T-155	-CH_3_	-CF_3_	(CH_3_)_2_CHCH_2_-	25.2 ± 1.4	53.7 ± 4.1	186 ± 7.5	97.6 ± 4.3
T-156	-C_6_H_5_	-CF_3_	(CH_3_)_2_CHCH_2_-	26.2 ± 1.1	31.2 ± 1.3	194 ± 7.1	105 ± 3.7
T-157	-C_10_H_7_	-CF_3_	(CH_3_)_2_CHCH_2_-	25.9 ± 3.1	15.3 ± 1.1	136 ± 4.3	119 ± 3.9
T-158	-C_6_H_5_	-CH_3_	(CH_3_)_2_CHCH_2_-	24.9 ± 1.4	27.2 ± 2.3	171 ± 7.6	148 ± 3.5
T-159	-CH_3_	-C_6_H_5_	(CH_3_)_2_CHCH_2_-	9.7 ± 1.5	14.9 ± 1.9	257 ± 8.5	189 ± 5.3
T-161	-C_4_H_3_S	-CF_3_	(CH_3_)_2_CHCH_2_-	46.3 ± 3.4	21.4 ± 2.1	116 ± 5.7	130 ± 5.5
T-162	-OCH_2_ C_6_H_5_	-CH_3_	(CH_3_)_2_CHCH_2_-	25.6 ± 1.9	30.7 ± 2.8	160 ± 7.8	117 ± 4.4
T-163	-NH-[2,4(CH_3_)_2_C_6_H_3_]	-CH_3_	(CH_3_)_2_CHCH_2_-	27.4 ± 4.2	53.5 ± 3.3	154 ± 6.4	84.5 ± 4.3
T-164	-CH_3_	-NH-[2,4(CH_3_)_2_C_6_H_3_]	(CH_3_)_2_CHCH_2_-	8.7 ± 1.2	32.7 ± 1.9	556 ± 13.2	134 ± 4.3
T-165	-HN-*p-*C_6_H_4_Cl	-CH_3_	(CH_3_)_2_CHCH_2_-	12.5 ± 2.3	24.3 ± 0.7	157 ± 6.1	165 ± 7.2
T-166	-CH_3_	-CH_3_	(CH_3_)_2_CHCH_2_-	45.7 ± 1.1	11.8 ± 1.6	159 ± 6.0	233 ± 8.8
T-167	-NHC_6_H_5_	-C_6_H_5_	(CH_3_)_2_CHCH_2_-	61.2 ± 3.9	74.8 ± 0.8	78.1 ± 2.8	136 ± 4.4
T-168	-C_4_H_3_O	-CF_3_	(CH_3_)_2_CHCH_2_-	69.4 ± 5.1	55.2 ± 1.3	70.0 ± 4.0	198 ± 5.7
T-169	-NHC_6_H_5_	-CH_3_	(CH_3_)_2_CHCH_2_-	78.3 ± 1.7	68.1 ± 3.1	56.9 ± 0.2	62.2 ± 3.3
T-170	-OCH_3_	-CH_3_	(CH_3_)_2_CHCH_2_-	76.9 ± 0.6	60.4 ± 0.9	33.3 ± 1.2	199 ± 10.2
Bnz				68.8 ± 1.7	51.4 ± 1.4	130.7 ± 8.8	191.3 ± 1.8
Nfx				76.6 ± 1.5	53.8 ± 1.3	70.4 ± 8.0	139.4 ± 3.0

**Table 2:** Estimated free energy of binding (FEB) and 2D-interaction profile representation for top scored (≤−8.3 kcal/mol) n-butyl and isobutyl quinoxaline-7-carboxylate 1,4-di-*N*-oxide derivatives binding on the active site of *Tc*TR.

2D-Interaction Profiles	
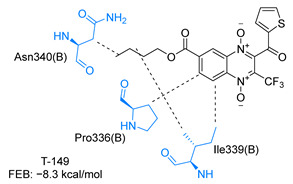	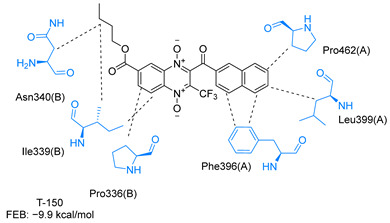
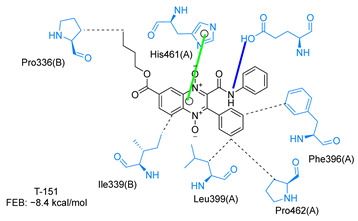	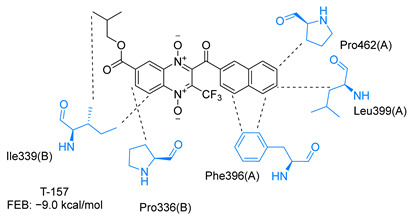
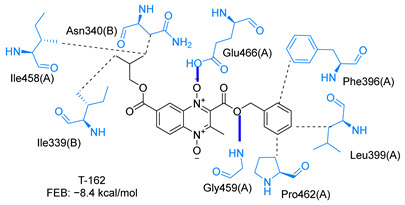	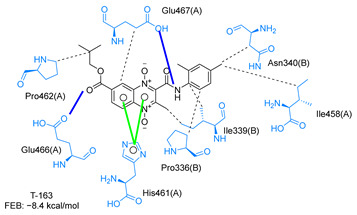
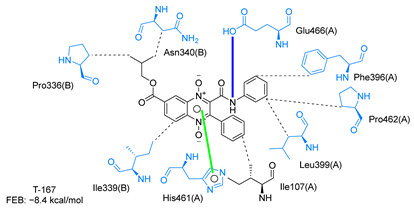	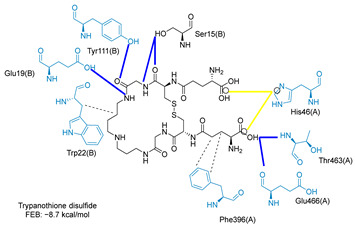

Interaction type legend: dashed grey line: hydrophobic interaction; solid blue line: hydrogen bond; solid golden line: salt bridge; solid green line: π-stacking.

**Table 3:** Inhibition of recombinant TR by n-butyl-quinoxaline-7-carboxylate 1,4-di-*N*-oxide derivatives.

Compound	Inhibitor [µM]	% Inhibition of TR	% Inhibition of TR
		44 µM [TS_2_]	100 µM [TS_2_]
T-147	20	35	28
	5	18	13
T-148	20	36	33
	5	18	16
T-150	20	insoluble	insoluble
	5	13	0

**Table 4:** hGR inhibition by the TR inhibitor T-147.

Substrate GSSG [µM]	% Inhibition of hGR	% Inhibition of hGR
	5 µM [T-147]	20 µM [T-147]
37	14	45
92	24	50

## Data Availability

Not applicable.

## Supplementary Materials

The supporting information can be downloaded at: https://www.mdpi.com/article/10.3390/ijms232113315/s1.
