# Supplementary material for: In Vitro and In Silico Analysis of New n-Butyl and Isobutyl Quinoxaline-7-carboxylate 1,4-di-N-oxide Derivatives against Trypanosoma cruzi as Trypanothione Reductase Inhibitors
Source: Int J Mol Sci. 2022 Nov 1;23(21):13315. doi: 10.3390/ijms232113315 (PMC9655728; doi:10.3390/ijms232113315)
Supplement: Supplementary file 1 [file ijms-23-13315-s001.zip › ijms-1916942-supplementary.pdf]

SUPPLEMENTARY INFORMATION

In vitro and *in silico* analysis of new n-butyl and isobutyl-quinoxaline-7-carboxylate 1,4-di-*N*-oxide derivatives against *Trypanosoma cruzi* as trypanothione reductase inhibitors

Alonzo González-González[1], Oscar Sánchez-Sánchez[1], R. Luise Krauth-Siegel[2], María Laura Bolognesi[3], Rogelio Gómez-Escobedo[4], Benjamín Nogueda-Torres[4], Lenci K. Vázquez-Jiménez[1], Emma Saavedra[5], Rusely Encalada[5], José Carlos Espinoza-Hicks[6], Alma D. Paz-González[1] and Gildardo Rivera[1*]

[1] Laboratorio de Biotecnología Farmacéutica, Centro de Biotecnología Genómica, Instituto Politécnico Nacional, Reynosa 88710, México

[2] Center of Biochemistry, Heidelberg University, Im Neuenheimer Feld 328, 69120 Heidelberg, Germany

[3] Department of Pharmacy and Biotechnology, Alma Mater Studiorum, University of Bologna, I-40126, Bologna, Italy

[4] Departamento de Parasitología, Escuela Nacional de Ciencias Biológicas Instituto Politécnico Nacional, Ciudad de México 07738, México

[5] Departamento de Bioquímica. Instituto Nacional de Cardiología Ignacio Chávez, Ciudad de México 14080, México

[6] Facultad de Ciencias Químicas, Universidad Autónoma de Chihuahua, Chihuahua 31125, México

*Corresponding author: gildardors@hotmail.com

NMR and IR data

Chemical compounds

T-137: 2-benzoyl-3-methyl-quinoxaline-7-butylcarboxylate-1,4-di-*N*-oxide. This compound obtained a 2.48 % yield. FT IR (ν cm^{-1}): 1325.37 (*N*-Oxide); 2900-3000 (ArC-H); 2900-2800 (C-H); 1721.02, 1673.79 (C=O); 1160-1300 ((C=O)-C-O) cm^{-1}. ^{1}H-NMR (400 MHz, DMSO-*d6*) δ ppm: 1H-NMR:CH_2-CH_2-CH_2-CH_3, t, 0.98, 3H; CH_2-CH_2-CH_2-CH_3, m, 1.48, 2H; CH_2-CH_2-CH_2-CH_3, m, 1.79, 2H; CH_2-CH_2-CH_2-CH_3, t, 4.43, 2H; C=O-phenyl, dd, 8.07, 2H; t, 7.78, 2H; t, 7.6, 2H; QX(C3) CH_3, s, 2.34, 3H; QX(ring), dd, 8.38, 1H; d, 8.51, 1H; ds, 9.01, 1H. ^{13}C-NMR (400 MHz, DMSO-*d6*) δ ppm: CH_2-CH_2-CH_2-CH_3, 14.02; CH_2-CH_2-CH_2-CH_3, 19.19; CH_2-CH_2-CH_2-CH_3, 30.61; CH_2-CH_2-CH_2-CH_3, 66.08; C=O-O, 164.64 C=O-phenyl, 129.90, 129.95, 134.59, 140.19; C=O, 187.42 QX(C3) CH_3, 14.30; QX (ring), 121.29, 121.68, 130.85, 133.44, 135.93, 138.45, 139.29, 14.01.

T-138: 2-(4-chlorophenyl) carbamoyl-3-methyl-quinoxaline-7-butylcarboxylate-1,4-di-*N*-oxide. This compound obtained a 4.39 % yield. FT IR (ν cm^{-1}): 1341.80 (*N*-Oxide), 3223.86 (N-H), 2900-3000 (ArC-H), 2900-2800 (C-H), 1720.41, 1676.46 (C=O); 1160-1300 ((C=O)-C-O); 600-830 (ArC-Cl). ^{1}H-NMR (400 MHz, DMSO-*d6*) δ ppm: 1H-NMR: CH_2-CH_2-CH_2-CH_3, t, 0.98, 3H; CH_2-CH_2-CH_2-CH_3, m, 1.47, 2H; CH_2-CH_2-CH_2-CH_3, m, 1.79, 2H; CH_2-CH_2-CH_2-CH_3, t, 4.42, 2H; C=O-NH-(4-Cl-phenyl), d, 7.71, 2H; d, 7.49, 2H; C=O-NH s,11.12, 1H; QX(C3) CH_3, s, 2.53, 3H; QX(ring), dd, 8.41, 1H; d, 8.62, 1H; ds, 9.01, 1H. ^{13}C-NMR (400 MHz, DMSO-*d6*) δ ppm: 13C-NMR: CH_2-CH_2-CH_2-CH_3, 13.78; CH_2-CH_2-CH_2-CH_3, 19.23; CH_2- CH_2-CH_2-CH_3, 30.68; CH_2-CH_2-CH_2-CH_3, 66.48; C=O-O, 163.64 C=O-NH-(4-chloro-phenyl), 129.03, 121.28; C=O, 156.02 QX(C3) CH_3, 15.09; QX(ring), 130.53, 131.21, 134.20, 135.05, 135.55, 138.04, 138.44, 143.21.

T-139: 2-benzoyl-3-trifluoromethyl-quinoxaline-7-butylcarboxylate-1,4-di-*N*-oxide. This compound obtained a 19.02 % yield. FT IR (ν cm^{-1}): 1338.87 (*N*-Oxide); 2900-3000 (ArC-H); 2900-2800 (C-H); 1724.48, 1689.38 (C=O); 1300-1160 ((C=O)-C-O); 1228-1135 (C-F). ^{1}H-NMR (400 MHz, DMSO-*d6*) δ ppm: 1H-NMR: CH_2-CH_2-CH_2-CH_3, t, 0.98, 3H; CH_2-CH_2-CH_2-CH_3, m, 1.48, 2H; CH_2-CH_2-CH_2-CH_3, m, 1.80, 2H; CH_2-CH_2-CH_2-CH_3, t, 4.42, 2H; C=O-phenyl, d, 8.50, 2H;t, 7.80, 2H; t, 7.6, 1H; QX(ring), dd, 8.14, 1H; d, 8.53, 1H; ds, 8.98, 1H. ^{13}C-NMR (400 MHz, DMSO-*d6*) δ ppm: 13C-NMR: CH_2-CH_2-CH_2-CH_3, 14.02; CH_2-CH_2-CH_2-CH_3, 19.18; CH_2-CH_2-CH_2-CH_3, 30.57; CH_2-CH_2-CH_2-CH_3, 66.29; C=O-O, 164.28; CF_3, 120.93; C=O-phenyl, 120.93, 129.71, 129.90, 130.17, 134.15, 134.40; C=O, 184.53; QX(ring), 121.50, 121.75, 130.52, 133.38, 138.96, 139.69, 141.21.

T-140: 2-(thienyl-2-carbonyl)-3-trifluoromethyl-quinoxaline-7-butylcarboxylate-1,4-di-*N*-oxide. This compound obtained a 20.58 % yield. FT IR (ν cm^{-1}): 1329.93 (*N*-Oxide); 2900-3000 (ArC-H); 2900-2800 (C-H); 1721.06, 1661.24 (C=O); 1300-1160 ((C=O)-C-O); 1227-1134 (C-F); 700-600 (ArC-S). ^{1}H-NMR (400 MHz, DMSO-*d6*) δ ppm: 1H-NMR: CH_2-CH_2-CH_2-CH_3, t, 0.98, 3H; CH_2-CH_2-CH_2-CH_3, m, 1.48, 2H; CH_2-CH_2-CH_2-CH_3, m, 1.80, 2H; CH_2-CH_2-CH_2-CH_3, t, 4.43, 2H; C=O-thiophenyl, m, 8.23, 2H; t, 7.32, 1H; QX(ring), dd, 8.30, 1H; d, 8.53, 1H; ds, 8.98, 1H. ^{13}C-NMR (400 MHz, DMSO-*d6*) δ ppm: 13C-NMR: CH_2-CH_2-CH_2-CH_3, 14.02; CH_2-CH_2-CH_2-CH_3, 19.18; CH_2-CH_2-CH_2-CH_3, 30.57; CH_2-CH_2-CH_2-CH_3, 66.29; C=O-O, 164.26; CF_3, 120.86; C=O-thiophenyl,134.20, 138.37, 141.29; C=O, 176.30; QX(ring), 121.64, 121.73, 129.90, 133.30, 138.43, 139.02, 139.61, 141.24.

T-141: 2-(tert-butoxy-carbonyl)-3-methyl-quinoxaline-7-butylcarboxylate-1,4-di-*N*-oxide. This compound obtained a 1.25 % yield. FT IR (ν cm^{-1}): 1334.51 (*N*-Oxide); 2900-3000 (ArC-H); 2900-2800 (C-H); 1723.75 (C=O); 1160-1300 ((C=O)-C-O). ^{1}H-NMR (400 MHz, DMSO-*d6*) δ ppm: 1H-NMR: CH_2-CH_2-CH_2-CH_3, t, 0.97, 3H; CH_2-CH_2-CH_2-CH_3, m, 1.46, 2H; CH_2-CH_2-CH_2-CH_3, m, 1.77, 2H; CH_2-CH_2-CH_2-CH_3, t, 4.41, 2H; C=O-O$(CH_3)_3$, s, 1.62, 9H; QX(C3) CH_3, s, 2.48, 3H; QX(ring), dd, 8.36, 1H; d, 8.53, 1H; ds, 8.92, 1H. ^{13}C-NMR (400 MHz, DMSO-*d6*) δ ppm: 13C-NMR (ppm): CH_2-CH_2-CH_2-CH_3, 14.01; CH_2-CH_2-CH_2-CH_3, 19.17; CH_2-CH_2-CH_2-CH_3, 30.60; CH_2-CH_2-CH_2-CH_3, 66.05; C=O-O, 164.56 C=O-O-t-but, 28.05, 86.28; C=O-O, 158.83 QX(C3) CH_3, 14.30; QX (ring), 121.41, 121.66, 130.99, 133.49, 136.90, 138.02, 138.02, 139.38.

T-142: 2-(metoxy-carbonyl)-3-methyl-quinoxaline-7-butylcarboxylate-1,4-di-*N*-oxide. This compound obtained a 1.69 % yield. FT IR (ν cm^{-1}): 1331.29 (*N*-Oxide); 2900-3000 (ArC-H); 2900-2800 (C-H); 1745.29, 1719.78 (C=O); 1160-1300 ((C=O)-C-O). ^{1}H-NMR (400 MHz, DMSO-*d6*) δ ppm: H-NMR: CH_2-CH_2-CH_2-CH_3, t, 0.97, 3H; CH_2-CH_2-CH_2-CH_3, m, 1.47, 2H; CH_2-CH_2-CH_2-CH_3, m, 1.77, 2H; CH_2-CH_2-CH_2-CH_3, t, 4.41, 2H; C=O-O(CH3), s, 4.04, 3H; QX(C3) CH_3, s, 2.46, 3H;

QX(ring), dd, 8.36, 1H; d, 8.53, 1H; ds, 8.92, 1H. ^{13}C-NMR (400 MHz, DMSO-*d6*) δ ppm: 13C-NMR (ppm): CH_2-CH_2-CH_2-CH_3, 14.01; CH_2-CH_2-CH_2-CH_3, 19.18; CH_2-CH_2-CH_2-CH_3, 30.60; CH_2-CH_2-CH_2-CH_3, 66.04; C=O-O, 164.53 C=O-O-met, 54.47; C=O-O, 160.49 QX(C3) CH_3, 14.61; QX (ring), 121.45, 121.63, 130.99, 131.03, 133.64, 138.13, 138.95, 139.90.

T-143: 2-(etoxy-carbonyl)-3-methyl-quinoxaline-7-butylcarboxylate-1,4-di-*N*-oxide. This compound obtained a 2.03 % yield. FT IR (ν cm^{-1}): 1331.29 (*N*-Oxide); 2900-3000 (ArC-H); 2900-2800 (C-H); 1745.29, 1719.78 (C=O); 1160-1300 ((C=O)-C-O). ^{1}H-NMR (400 MHz, DMSO-*d6*) δ ppm: H-NMR: CH_2-CH_2-CH_2-CH_3, t, 0.97, 3H; CH_2-CH_2-CH_2-CH_3, m, 1.46, 2H; CH_2-CH_2-CH_2-CH_3, m, 1.76, 2H; CH_2-CH_2-CH_2-CH_3, t, 4.40, 2H; C=O-O(CH_2CH_3), t, 1.38, 3H; C=O-O(CH_2CH_3), q, 4.53, 2H; QX(C3) CH_3, s, 2.48, 3H; QX(ring), dd, 8.37, 1H; d, 8.53, 1H; ds, 8.93, 1H. ^{13}C-NMR (400 MHz, DMSO-*d6*) δ ppm: 13C-NMR (ppm): CH_2-CH_2-CH_2-CH_3, 13.73; CH_2-CH_2-CH_2-CH_3, 19.23; CH_2-CH_2-CH_2-CH_3, 30.64; CH_2-CH_2-CH_2-CH_3, 66.22; C=O-O, 164.33; C=O-O(CH_2CH_3), 14.44; C=O-O(CH_2CH_3), 63.88; QX(C3):CH_3: 14.01 C=O-O, 159.57; QX(ring), 120.78, 120.99, 122.32, 122.54, 131.35, 132.44, 132.45, 134.42

T-144: 2-(2,4-dimethyl-phenyl) carbamoyl-3-methyl-quinoxaline-7-butylcarboxylate-1,4-di-*N*-oxide. This compound obtained a 2.68 % yield. FT IR (ν cm^{-1}): 1318.34 (*N*-Oxide); 3362.94 (N-H); 2900-3000 (ArC-H); 2900-2800 (C-H); 1720.34, 1694.60 (C=O); 1160-1300 ((C=O)-C-O). ^{1}H-NMR (400 MHz, DMSO-*d6*) δ ppm: 1H-NMR: CH_2-CH_2-CH_2-CH_3, t, 0.98, 3H; CH_2-CH_2-CH_2-CH_3, m, 1.48, 2H; CH_2-CH_2-CH_2-CH_3, m, 1.79, 2H; CH_2-CH_2-CH_2-CH_3, t, 4.40, 2H; C=O-NH-(2,4-dimethyl-phenyl), d, 7.51, 1H; d, 7.09, 1H; s, 7.12, 1H; C=O-NH-(2,4-dimethyl-phenyl) s, 2.51, 3H; s, 2.30, 3H; C=O-NH s,10.24, 1H; QX(C3) CH_3, s, 2.31, 3H; QX(ring), dd, 8.41, 1H; d, 8.64, 1H; ds, 9.01, 1H. ^{13}C-NMR (400 MHz, DMSO-*d6*) δ ppm: 13C-NMR: CH_2-CH_2-CH_2-CH_3, 14.02; CH_2-CH_2-CH_2-CH_3, 19.19; CH_2-CH_2-CH_2-CH_3, 30.66; CH_2-CH_2-CH_2-CH_3, 66.08; C=O-O, 164.58 C=O-NH-(2,4-dimethyl-phenyl), 125.09, 127.25, 131.62, 132.11, 132.49, 135.93; C=O-NH-(2,4-dimethyl-phenyl) 18.17, 20.87; C=O, 157.84 QX(C3) CH_3, 14.73; QX(ring), 121.62, 121.68, 130.40, 131.07, 133.52, 137.55, 139.03, 139.70.

T-145: 2-(methyl-carbonyl)-3-methyl-quinoxaline-7-butylcarboxylate-1,4-di-*N*-oxide. This compound obtained a 2.37 % yield. FT IR (ν cm^{-1}): 1323.15 (*N*-Oxide); 2900-3000 (ArC-H); 2900-2800 (C-H); 1727.03, 1700.97 (C=O); 1160-1300 ((C=O)-C-O). ^{1}H-NMR (400 MHz, DMSO-*d6*) δ ppm: 1H-NMR: CH_2-CH_2-CH_2-CH_3, t, 0.98, 3H; CH_2-CH_2-CH_2-CH_3, m, 1.47, 2H; CH_2-CH_2-CH_2-CH_3, m, 1.78, 2H; CH_2-CH_2-CH_2-CH_3, t, 4.40, 2H; C=O-methyl, s, 2.67, 3H; QX(C3) CH_3, s, 2.40, 3H; QX(ring), dd, 8.41, 1H; d, 8.64, 1H; ds, 9.01, 1H. ^{13}C-NMR (400 MHz, DMSO-*d6*) δ ppm: 13C-NMR: CH_2-CH_2-CH_2-CH_3, 14.02; CH_2-CH_2-CH_2-CH_3, 19.18; CH_2-CH_2-CH_2-CH_3, 30.60; CH_2-CH_2-CH_2-CH_3, 66.06; C=O-O, 164.58; C=O-methyl, 29.96; C=O, 195.61;QX(ring), 121.22, 121.58, 130.93, 133.48, 137.86, 138.88, 139.73, 141.15

T-146: 2-(tert-butyl-carbonyl)-3-trifluoromethyl-quinoxaline-7-butylcarboxylate-1,4-di-*N*-oxide. This compound obtained a 1.02 % yield. FT IR (ν cm^{-1}): 1320 (*N*-Oxide); 2900-3000 (ArC-H); 2900-2800 (C-H); 1709.65 (C=O); 1300-1160 ((C=O)-C-O); 1247-1154 (C-F). ^{1}H-NMR (400 MHz, DMSO-*d6*) δ ppm: 1H-NMR: CH_2-CH_2-CH_2-CH_3, t, 0.97, 3H; CH_2-CH_2-CH_2-CH_3, m, 1.47, 2H; CH_2-CH_2-CH_2-CH_3, m, 1.78, 2H; CH_2-CH_2-CH_2-CH_3, t, 4.42, 2H; C=O-t-butyl, s, 1.24, 9H; QX(ring), dd, 8.45, 1H; d, 8.62, 1H; ds, 8.90, 1H. ^{13}C-NMR (400 MHz, DMSO-*d6*) δ ppm: 13C-NMR: CH_2-CH_2-CH_2-CH_3, 14.01; CH_2-CH_2-CH_2-CH_3, 19.17; CH_2-CH_2-CH_2-CH_3, 30.57; CH_2-CH_2-CH_2-CH_3, 66.22; C=O-O, 164.34; QX(ring), 121.74, 121.77, 130.72, 132.16, 134.32, 139.65, 141.15.

T-147: 2-(2-naphthyl-carbonyl)-3-trifluoromethyl-quinoxaline-7-butylcarboxylate-1,4-di-*N*-oxide. This compound obtained a 1.17 % yield. FT IR (ν cm^{-1}): 1337.80 (*N*-Oxide); 2900-3000 (ArC-H); 2900-2800 (C-H); 1725.93, 1674.96 (C=O); 1300-1160 ((C=O)-C-O); 1259-1156 (C-F). ^{1}H-NMR (400 MHz, DMSO-*d6*) δ ppm: 1H-NMR: CH_2-CH_2-CH_2-CH_3, t, 0.99, 3H; CH_2-CH_2-CH_2-CH_3, m, 1.50, 2H; CH_2-CH_2-CH_2-CH_3, m, 1.80, 2H; CH_2-CH_2-CH_2-CH_3, t, 4.45, 2H; C=O-naphthyl, t, 7.75, 1H; t, 7.65, 1H; d, 8.06, 1H; d, 8.01, 1H; d, 9.03, 1H; ds, 8.14, 1H; QX(ring), dd, 8.53, 1H; d, 8.62, 1H; ds, 8.95, 1H. ^{13}C-NMR (400 MHz, DMSO-*d6*) δ ppm: 13C-NMR: CH_2-CH_2-CH_2-CH_3, 14.02; CH_2-CH_2-CH_2-CH_3, 19.19; CH_2-CH_2-CH_2-CH_3, 30.58; CH_2-CH_2-CH_2-CH_3, 66.32; C=O-O, 164.28; CF_3, C=O-naphthyl,123.45, 127.95, 128.45, 129.71, 131.32, 131.89, 134.21, 136.51; C=O, 184.40; QX(ring), 121.63, 121.80, 130.11, 132.82, 133.14, 139.09, 139.65, 141.27.

T-148: 2-(benzyloxy-carbonyl)-3-methyl-quinoxaline-7-butylcarboxylate-1,4-di-*N*-oxide. This compound obtained a 1.38 % yield. FT IR (ν cm^{-1}): 1331.49 (*N*-Oxide); 2900-3000 (ArC-H); 2900-2800 (C-H); 1749.31, 1719.50 (C=O); 1300-1160 ((C=O)-C-O). ^{1}H-NMR (400 MHz, DMSO-*d6*) δ ppm: 1H-NMR: CH_2-CH_2-CH_2-CH_3, t, 0.97, 3H; CH_2-CH_2-CH_2-CH_3, m, 1.47, 2H; CH_2-CH_2-CH_2-CH_3, m, 1.77, 2H; CH_2-CH_2-CH_2-CH_3, t, 4.40, 2H; C=O-O(CH_2)-phenyl, m, 7.6-7.1, 5H; C=O-

O(CH_2)-phenyl, s, 5,56, 2H; QX(C3) CH_3, s, 2.42, 3H; QX(ring), dd, 8.37, 1H; d, 8.55, 1H; ds, 8.93, 1H. ^{13}C-NMR (400 MHz, DMSO-*d6*) δ ppm: 13C-NMR (ppm): CH_2-CH_2-CH_2-CH_3, 13.74; CH_2-CH_2-CH_2-CH_3, 19.23; CH_2-CH_2-CH_2-CH_3, 30.64; CH_2-CH_2-CH_2-CH_3, 66.22; C=O-O, 164.32 C=O-O-CH_2-phenyl 69.34, C=O-O-CH_2-phenyl, 121.07; C=O-O, 159.47 QX(C3) CH_3, 14.26; QX (ring), 130.89, 131.33, 132.44, 133.98, 134.43, 137.79, 138.71, 139.77.

T-153: 2-(methyl-carbonyl)-3-trifluoromethyl-quinoxaline-7-butylcarboxylate-1,4-di-*N*-oxide. This compound obtained a 6.72 % yield. FT IR (ν cm^{-1}): 1339.19 (*N*-Oxide); 2900-3000 (ArC-H); 2900-2800 (C-H); 1725.95, 1713.52 (C=O); 1160-1300 ((C=O)-C-O) 1234-1176 (C-F). ^{1}H-NMR: CH_2-CH_2-CH_2-CH_3, t, 0.98, 3H; CH_2-CH_2-CH_2-CH_3, m, 1.48, 2H; CH_2-CH_2-CH_2-CH_3, m, 1.78, 2H; CH_2-CH_2-CH_2-CH_3, t, 4.40, 2H; C=O-methyl, s, 2.63, 3H; QX(ring), dd, 8.52, 1H; d, 8.59, 1H; ds, 8.93, 1H. ^{13}C-NMR (400 MHz, DMSO-*d6*) δ ppm: 13C-NMR (ppm): CH_2-CH_2-CH_2-CH_3, 14.01; CH_2-CH_2-CH_2-CH_3, 19.18; CH_2-CH_2-CH_2-CH_3, 30.56; CH_2-CH_2-CH_2-CH_3, 66.28; C=O-O, 164.21 C=O-CH_3, 29.64; QX(ring), 121.41, 121.65, 133.45, 134.14, 138.96, 140.13, 140.80.

T-154: 2-(etoxy-carbonyl)-3-trifluoromethyl-quinoxaline-7-butylcarboxylate-1,4-di-*N*-oxide. This compound obtained a 6.21 % yield. FT IR (ν cm^{-1}): 1346.81 (*N*-Oxide); 2900-3000 (ArC-H); 2900-2800 (C-H); 1744.39, 1729.69 (C=O); 1160-1300 ((C=O)-C-O), 1225-1156 (C-F). ^{1}H-NMR: CH_2-CH_2-CH_2-CH_3, t, 0.98, 3H; CH_2-CH_2-CH_2-CH_3, m, 1.48, 2H; CH_2-CH_2-CH_2-CH_3, m, 1.78, 2H; CH_2-CH_2-CH_2-CH_3, t, 4.42, 2H; C=O-O(CH_2CH_3), t, 1.36, 3H; C=O-O(CH_2CH_3), q, 4.51, 2H; QX(ring), dd, 8.51, 1H; d, 8.57, 1H; ds, 8.90, 1H. ^{13}C-NMR (400 MHz, DMSO-*d6*) δ ppm: 13C-NMR (ppm): CH_2-CH_2-CH_2-CH_3, 14.00; CH_2-CH_2-CH_2-CH_3, 19.17; CH_2-CH_2-CH_2-CH_3, 30.56; CH_2-CH_2-CH_2-CH_3, 66.27; C=O-O, 164.20; C=O-O(CH_2CH_3), 14.07; C=O-O(CH_2CH_3), 64.27; C=O-O, 158.13; QX(ring), 120.70, 121.65, 121.74, 133.50, 134.35, 135.03, 139.39, 140.87.

T-155. 2-(methyl-carbonyl)-3-trifluoromethyl-quinoxaline-7-isobutylcarboxylate-1,4-di-*N*-oxide. This compound obtained a: 4.8 % yield. FT IR (ν cm^{-1}): 1334 (N-Oxide), 2962-3093 (ArC-H), 1721 (C=O), 1282-1152 (Ar-CF_3) cm^{-1}. OSS-001. 1H-NMR (400 MHz, DMSO-d6) δ ppm: $CH_2CH(CH_3)_2$, d, 1.02, 6H; $CH_2CH(CH_3)_2$, m, 2.10, 1H; $CH_2CH(CH_3)_2$, d, 4.21, 2H; C=O-CH_3, s, 2.63, 3H; QX (ring), dd, 8.53, 1H; d 8.59, 1H; ds, 8.93, 1H. 13C-RMN (400 MHz, DMSO-d6) δ ppm: $CH_2CH(CH_3)_2$, 19.32; $CH_2CH(CH_3)_2$, 29.65; $CH_2CH(CH_3)_2$, 72.15; CF_3- QX(ring), 121.46, 121.64, 133.46, 134.10, 138.98, 140.14, 140.98; C=O-CH3, 27.81; C=O-O-, 164.16; C=O-CH_3, 191.77.

T-156. 2-(phenyl-carbonyl)-3-trifluoromethyl-quinoxaline-7-isobutylcarboxylate-1,4-di-*N*-oxide. This compound obtained a: 2.7 % yield. FT IR (ν cm^{-1}): 1338 (N-Oxide), 2876-3095 (ArC-H), 1721, 1689 (C=O), 1285-1132 (Ar-CF_3) cm^{-1}. 1H-RMN (400MHz, DMSO-d6) δ ppm: $CH_2CH(CH_3)_2$, d, 1.03, 6H; $CH_2CH(CH_3)_2$, m, 2.18, 1H; $CH_2CH(CH_3)_2$, d, 4.25, 2H; C=O-phenyl, t, 7.47, 1H; d, 7.64, 2H; d, 7.81, 2H; QX (ring), d, 8.16, 1H; d, 8.52, 1H; s, 8.99, 1H. 13C-RMN (400MHz, DMSO-d6) δ ppm: $CH_2CH(CH_3)_2$, 19.05; $CH_2CH(CH_3)_2$, 27.83; $CH_2CH(CH_3)_2$, 72.15; CF_3- QX(ring), 121.55, 121.74, 127.68, 129.21, 129.90, 133.38, 135.90, 141.25.; C=O-O-, 164.23; C=O-phenyl, 184.52.

T-157. 2-(naphthyl-carbonyl)-3-trifluoromethyl-quinoxaline-7-isobutylcarboxylate-1,4-di-*N*-oxide. This compound obtained a: 5.0 % yield. FT IR (ν cm^{-1}):1339 (N-Oxide), 2875-3095 (ArC-H), 1722, 1687 (C=O), 1339, 1170-1125 (Ar-CF_3) cm^{-1}. 1H-RMN (400 MHz, DMSO-d6) δ ppm: $CH_2CH(CH_3)_2$, d, 1.04, 6H; $CH_2CH(CH_3)_2$, m, 2.14, 1H; $CH_2CH(CH_3)_2$, d, 4.24, 2H; C=O-naphthyl, s, 8.85, 1H; d, 8.86, 1H; d, 8.54, 1H; d, 8.08, 1H; d, 8.01, 1H; t, 7.75, 1H; t, 7.68, 1H; QX (ring), d, 8.14, 1H; d, 8.57 1H; s, 9.04, 1H. . 13C-RMN (400 MHz, DMSO-d6) δ ppm: $CH_2CH(CH_3)_2$, 19.32; $CH_2CH(CH_3)_2$, 27.84; $CH_2CH(CH_3)_2$, 72.16; CF_3- QX(ring),121.68, 121.90, 123.45, 127.95, 129.78, 132.82, 134.18, 141.29; C=O-O-, 164.24; C=O-naphthyl, 184.39.

T-158. 2-(phenyl-carbonyl)-3-methyl-quinoxaline-7-isobutylcarboxylate-1,4-di-*N*-oxide. This compound obtained a: 20 % yield FT IR (ν cm^{-1}): 1322 (N-Oxide), 2970-3101 (ArC-H), 1726, 1675 (C=O), 1322 cm^{-1}. 1H-RMN (400 MHz, DMSO-d6) δ ppm: CH2CH(CH3)2, d, 1.04, 6H; CH2CH(CH3)2, m, 2.34, 1H; CH2CH(CH3)2, d, 4.22, 2H; CH3-QX, s, 2.46, 3H; C=O-phenyl, t, 7.58, 3H; d, 8.10, 2H; d, 8.45, 2H; QX (ring), d, 8.64, 1H; d, 8.86, 1H; s, 9.00, 1H. 13C-RMN (400 MHz, DMSO-d6) δ ppm: CH2CH(CH3)2, 19.34; CH2CH(CH3)2, 27.85; CH2CH(CH3)2, 71.96; CH3-QX (ring), 14.30; QX(ring), 121.34, 121.68, 130.84, 133.40, 134.55, 135.92, 138.47, 139.31; C=O-O-, 164.60; C=O-phenyl, 187.42.

T-159. 2-methyl-3-(phenyl-carbonyl)-quinoxaline-7-isobutylcarboxylate-1,4-di-*N*-oxide. This compound obtained a: 10.5 % yield. FT IR (ν cm^{-1}): 1322 (N-Oxide), 2874-3110 (ArC-H), 1718, 1685 (C=O) cm^{-1}. 1H-RMN (400 MHz, DMSO-d6) δ ppm: $CH_2CH(CH_3)_2$, d, 1.05, 6H; $CH_2CH(CH_3)_2$, m, 2.34, 1H; $CH_2CH(CH_3)_2$, d, 4.23, 2H; QX-phenyl, t, 7.60, 1H; d, 7.58, 2H; d, 8.10, 2H; QX (ring), d, 8.41, 1H; d, 8.53, 1H; s, 9.03, 1H. 13C-RMN (400 MHz, DMSO-d6) δ ppm: $CH_2CH(CH_3)_2$, 19.45; $CH_2CH(CH_3)_2$, 27.91; $CH_2CH(CH_3)_2$, 72.00; C=O-CH_3, 30.20; C=O-O-, 164.19; C=O-CH_3, 187.42; QX(ring), 121.35, 121.54, 128.97, 130.39, 131.58, 133.52, 135.91, 140.43.

T-161. 2-(thienyl-2-carbonyl)-3-trifluoromethyl-quinoxaline-7-isobutylcarboxylate-1,4-di-*N*-oxide. This compound obtained a: 4.1 % yield. FT IR (ν cm^{-1}): 1358 (N-Oxide), 2959 (ArC-H), 1720, 1657 (C=O), 1155 (Ar-CF_3) cm^{-1}. OSS-015. 1H-RMN (400 MHz, DMSO-d6) δ ppm: $CH_2CH(CH_3)_2$, d, 1.05, 6H; $CH_2CH(CH_3)_2$, m, 2.18, 1H; $CH_2CH(CH_3)_2$, d, 4.24, 2H; C=O-thienyl, s, 7.32, 1H; ds, 8.22-8.30, 2H; QX (ring), m, 8.58, 2H; s, 8.99, 1H. 13C-RMN (400 MHz, DMSO-d6) δ ppm: $CH_2CH(CH_3)_2$, 19.32; $CH_2CH(CH_3)_2$, 27.83; $CH_2CH(CH_3)_2$, 72.15; CF_3- QX(ring), 121.69, 121.73, 129.92, 133.38, 134.16, 138.43, 139.02, 139.63; C=O-O-, 164.22; C=O-thiophenyl, 176.29.

T-162. 2-(benzyloxy-carbonyl)-3-methyl-quinoxaline-7-isobutylcarboxylate-1,4-di-*N*-oxide. This compound obtained a: 4.0 % yield. FT IR (ν cm^{-1}): 1330 (N-Oxide), 2960-2892 (ArC-H), 1752, 1723 (C=O) cm^{-1}. ^{1}H-NMR (400 MHz, DMSO-*d6*) δ ppm: 1H: $CH_2CH(CH_3)_2$, d, 1.06, 6H; $CH_2CH(CH_3)_2$, m, 2.15, 1H; $CH_2CH(CH_3)_2$, d, 4.23, 2H; C=O-O(CH_2)-phenyl, t, 7.25 1H, t, 7.42 2H, d, 7.51 2H; C=O-O(CH_2)-phenyl, s, 5,56, 2H; QX(C3) CH_3, s, 2.50, 3H; QX(ring), d, 8.45, 1H; d, 8.65, 1H; s, 9.25, 1H ^{13}C-NMR (400 MHz, DMSO-*d6*) δ ppm: 13C-NMR (ppm): $CH_2CH(CH_3)_2$, 19.16; $CH_2CH(CH_3)_2$, 27.87; $CH_2CH(CH_3)_2$, 72.26; C=O-O, 164.26 C=O-O-CH_2-phenyl 69.34, C=O-O-CH_2-phenyl, 121.08, 122.30, 128, 128.80 ; C=O-O, 159.47 QX(C3) CH_3, 14.20; QX (ring), 137.8, 134.45, 133.98, 131.34, 129.16, 128.90, 128.87.

T-163. 2-(2,4-dimethylphenyl-carbamoyl)-3-methyl-quinoxaline-7-isobutylcarboxylate-1,4-di-*N*-oxide. This compound obtained a: 3.7 % yield. FT IR (ν cm^{-1}):1374 (N-Oxide), 3349 (N-H), 2957-2873 (ArC-H), 1717, 1695 (C=O) cm^{-1}. ^{1}H-NMR (400 MHz, DMSO-*d6*) δ ppm: 1H: $CH_2CH(CH_3)_2$, d, 1.08, 6H; $CH_2CH(CH_3)_2$, m, 2.18, 1H; $CH_2CH(CH_3)_2$, d, 4.24, 2H; C=O-NH-(4-Cl-phenyl), d, 7.88, 2H; d, 7.99, 2H; C=O-NH s,10.74, 1H; QX(C3) CH_3, s, 2.43, 3H; QX(ring), d, 8.45, 1H; d, 8.69, 1H; s, 9.26, 1H. ^{13}C-NMR (400 MHz, DMSO-*d6*) δ ppm: $CH_2CH(CH_3)_2$, 19.18; $CH_2CH(CH_3)_2$, 27.86; $CH_2CH(CH_3)_2$, 72.26; C=O-O, 164.22 C=O-NH-(2,4-dimethyl-phenyl), 123.11, 127.34, 131.64, 134.39, 135.91, 137.37; C=O-NH-(2,4-dimethyl-phenyl) 18.11, 20.98; C=O, 159.90 QX(C3) CH3, 15.83; QX(ring), 121.22, 122.34, 129.74, 131.41, 132.20, 137.14, 138.20, 144.50.

T-164. 2-methyl-3-(2,4-dimethylphenyl-carbamoyl)-quinoxaline-7-isobutylcarboxylate-1,4-di-N-oxide. This compound obtained a: 2.3 % yield. FT IR (ν cm^{-1}): 1335 (N-Oxide), 3103 (N-H), 2960-2872 (ArC-H), 1725, 1664 (C=O) 1335 cm^{-1}^{1}H-NMR (400 MHz, DMSO-*d6*) δ ppm: 1H: $CH_2CH(CH_3)_2$, d, 1.11, 6H; $CH_2CH(CH_3)_2$, m, 2.18, 1H; $CH_2CH(CH_3)_2$, d, 4.24, 2H; C=O-NH-(4-Cl-phenyl), d, 7.08, 2H; d, 7.88, 2H; C=O-NH s,9.94, 1H; QX(C2) CH_3, s, 2.36, 3H; QX(ring), d, 8.17, 1H; d, 8.39, 1H; s, 8.83, 1H. ^{13}C-NMR (400 MHz, DMSO-*d6*) δ ppm: $CH_2CH(CH_3)_2$, 19.23; $CH_2CH(CH_3)_2$, 27.92; $CH_2CH(CH_3)_2$, 71.86; C=O-O, 165.58 C=O-NH-(2,4-dimethyl-phenyl), 128.78, 129.44, 131.29, 132.94, 133.29, 140.89; C=O-NH-(2,4-dimethyl-phenyl) 17.78, 20.95; C=O, 161.67 QX(C2) CH_3, 25.12; QX(ring), 121.95, 127.44, 130.89, 131.19 131.79, 134.95, 142.41, 144.66.

T-165. 2-(*p*-chlorophenyl-carbamoyl)-3-methyl-quinoxaline-7-isobutylcarboxylate-1,4-di-*N*-oxide. This compound obtained a: 14 % yield. FT IR (ν cm^{-1}): 1342 (N-Oxide), 3245-3188 (N-H), 2965-2873 (ArC-H), 1722, 1685 (C=O), 829 (Ar-Cl) cm^{-1}. 1H-RMN (400 MHz, DMSO-d6) δ ppm: $CH_2CH(CH_3)_2$, d, 1.05, 6H; $CH_2CH(CH_3)_2$, m, 2.16, 1H; $CH_2CH(CH_3)_2$, d, 4.22, 2H; CH_3-QX, s, 2.53, 3H; C=O-NH-4-Cl-phenyl, d, 7.58, 2H; d, 7.69, 2H; QX (ring), d, 8.44, 1H; d, 8.62, 1H; s, 9.02, 1H; C=O-NH-, s, 11.12, 1H. . 13C-RMN (400 MHz, DMSO-d6) δ ppm: $CH_2CH(CH_3)_2$, 19.36; $CH_2CH(CH_3)_2$, 27.84; $CH_2CH(CH_3)_2$, 72.00; CH_3-QX (ring), 14.71; C=O-O$CH_2CH(CH_3)_2$, 157.09; C=O-NH-, 164.50; QX(ring), 121.66, 121.73, 128.95, 129.58, 131.21, 133.64, 139.95, 140.47.

T-166. 2-(methyl-carbonyl)-3-methyl-quinoxaline-7-isobutylcarboxylate-1,4-di-*N*-oxide. This compound obtained a: 13.4 % yield. FT IR (ν cm^{-1}): 1328 (N-oxide), 2962 (ArC-H), 1716 (C=O) cm^{-1}. 1H-RMN (400 MHz, DMSO-d6) δ ppm: $CH_2CH(CH_3)_2$, d, 1.04, 6H; $CH_2CH(CH_3)_2$, m, 2.17, 1H; $CH_2CH(CH_3)_2$, d, 4.21, 2H; CH_3-QX (ring), s, 2.67, 3H; C=O-CH3, s, 2.40, 3H; QX (ring), d, 8.40, 1H; d 8.58, 1H; s, 8.96, 1H. 13C-RMN (400 MHz, DMSO-d6) δ ppm: $CH_2CH(CH_3)_2$, 14.00;

$CH_2CH(CH_3)_2$, 19.34; $CH_2CH(CH_3)_2$, 71.96; CH_3-QX (ring), 27.83; C=O-CH_3, 29.96; C=O-O-, 164.52; C=O-CH_3, 195.61; QX(ring), 121.26, 121.58, 130.92, 133.45, 137.87, 138.91, 139.75, 141.16.

T-167. 2-(phenyl-carbamoyl)-3-phenyl-quinoxaline-7-isobutylcarboxylate-1,4-di-*N*-oxide. This compound obtained a: 3.5 % yield. FT IR (ν cm^{-1}): 1333 (N-Oxide), 3198 (N-H), 2961 (ArC-H), 1723, 1685 (C=O), cm^{-1}. 1H-RMN (400 MHz, DMSO-d6) δ ppm: $CH_2CH(CH_3)_2$, d, 1.05, 6H; $CH_2CH(CH_3)_2$, m, 2.16, 1H; $CH_2CH(CH_3)_2$, d, 4.24, 2H; QX-phenyl, t, 7.23, 1H; d, 7.38, 2H; d, 7.33, 2H; C=O-NH-phenyl, t, 7.17, 1H; d, 7.62, 2H; d, 7.31,2H; QX (ring), d, 8.51, 1H; d, 8.69, 1H; s, 9.06, 1H; C=O-NH-, s, 10.79, 1H. . 13C-RMN (400 MHz, DMSO-d6) δ ppm: $CH_2CH(CH_3)_2$, 19.35; $CH_2CH(CH_3)_2$, 27.84; $CH_2CH(CH_3)_2$, 72.02; C=O-O-, 156.70; C=O-NH-, 164.49; QX(ring), 121.76, 122.28, 128.71, 129.47, 130.25, 133.70, 137.97, 139.53.

T-168. 2-(furyl-carbonyl)-3-trifluoromethyl-quinoxaline-7-isbutylcarboxylate-1,4-di-*N*-oxide. This compound obtained a: 9.0 % yield. FT IR (ν cm^{-1}): 1339 (N-Oxide), 2962-2876 (ArC-H), 1722, 1665 (C=O), cm^{-1}. 1H-RMN (400 MHz, DMSO-d6) δ ppm: $CH_2CH(CH_3)_2$, d, 1.05, 6H; $CH_2CH(CH_3)_2$, m, 2.13, 1H; $CH_2CH(CH_3)_2$, d, 4.24.2H; C=O-furyl, s, 6.84, 1H; s, 7.89, 1H; s, 8.24, 1H; QX (ring), c, 8.59, 2H; s, 8.99, 1H. 13C-RMN (400 MHz, DMSO-d6) δ ppm: $CH_2CH(CH_3)_2$, 19.34; $CH_2CH(CH_3)_2$, 27.82; $CH_2CH(CH_3)_2$, 72.16; CF_3- QX(ring), 121.71, 121.75, 124.62, 133.44, 134.16, 137.74, 139.61, 141.25; C=O-O-, 164.19; C=O-furyl, 170.16.

T-169. 2-(phenyl-carbamoyl)-3-methyl-quinoxaline-7-isobutylcarboxylate-1,4-di-*N*-oxide. This compound obtained a: 22.5 % yield. FT IR (ν cm^{-1}): 3261 cm^{-1}:1375 (N-Oxide), 3260 (N-H,), 2960-2874 (ArC-H), 1720, 1677 (C=O) cm^{-1}. 1H-RMN (400MHz, DMSO-d6) δ ppm: $CH_2CH(CH_3)_2$, d, 1.05, 6H; $CH_2CH(CH_3)_2$, m, 2.16, 1H; $CH_2CH(CH_3)_2$, d, 4.23, 2H; CH3-QX, s, 2.54, 3H; C=O-NH-phenyl, t, 7.57, 1H; d, 7.41, 2H; d, 7.69, 1H; QX (ring), d, 8.44, 1H; d, 8.65, 1H; s, 9.03, 1H; C=O-NH-, s, 10.96, 1H. 13C-RMN (400 MHz, DMSO-d6) δ ppm: $CH_2CH(CH_3)_2$, 19.36; $CH_2CH(CH_3)_2$, 14.72; $CH_2CH(CH_3)_2$, 27.84; CH_3-QX (ring), 14.91; C=O-O-, 157.30; C=O-NH-, 164.52; QX(ring), 121.65, 121.72, 125.24, 129.63, 131.16, 133.58, 138.27, 140.42.

T-170. 2-(methoxy-carbonyl)-3-methyl-quinoxaline-7-isobutylcarboxylate-1,4-di-*N*-oxide. This compound obtained a: 4.3 % yield. FT IR (ν cm^{-1}): 1327 (N-oxide), 2959-3099 (ArC-H), 1744, 1718 (C=O), 1327 cm^{-1}. ^{1}H-RMN (400 MHz, DMSO-d6) δ ppm: $CH_2CH(CH_3)_2$, d, 1.08, 6H; $CH_2CH(CH_3)_2$, m, 2.20, 1H; $CH_2CH(CH_3)_2$, d, 4.23, 2H; CH_3-QX (ring), s, 2.48, 3H; C=O(OCH_3), s, 4.08, 3H; QX (ring), d, 8.42, 1H; d, 8.59, 1H; s, 8.92, 1H. ^{13}C-NMR (400 MHz, DMSO-d6) δ ppm: $CH_2CH(CH_3)_2$, 14.61; $CH_2CH(CH_3)_2$, 19.32; $CH_2CH(CH_3)_2$, 71.97; CH_3-QX (ring), 27.83; C=O-O-CH_3, 54.47; C=O-O-CH_3, 160.46; C=O-O-, 164.48; QX(ring), 121.44, 121.63, 131.02, 132.10, 132.65, 135.60, 138.15, 139.90.

T-171: 2-phenyl carbamoyl-3-phenyl-quinoxaline-7-butylcarboxylate-1,4-di-*N*-oxide. This compound obtained a 21.88 % yield. FT IR (ν cm^{-1}): 1337.66 (*N*-Oxide); 3297.34 (N-H); 2900-3000 (ArC-H); 2900-2800 (C-H); 1718.67, 1665.84 (C=O); 1160-1300 ((C=O)-C-O). ^{1}H-NMR: CH_2-CH_2-CH_2-CH_3, t, 0.98, 3H; CH_2-CH_2-CH_2-CH_3, m, 1.49, 2H; CH_2-CH_2-CH_2-CH_3, m, 1.79, 2H; CH_2-CH_2-CH_2-CH_3, t, 4.43, 2H; C=O-NH-(phenyl), dd, 7.56, 2H; t, 7.35, 2H; t, 7.12, 1H; C=O-NH s,10.79, 1H; QX(ring), dd, 8.48, 1H; d, 8.70, 1H; ds, 9.04, 1H. ^{13}C-NMR (400 MHz, DMSO-*d6*) δ ppm: 13C-NMR (ppm): CH_2-CH_2-CH_2-CH_3, 14.03; CH_2-CH_2-CH_2-CH_3, 19.19; CH_2-CH_2-CH_2-CH_3, 30.61; CH_2-CH_2-CH_2-CH_3, 66.12; C=O-O, 164.54; C=O-NH-(phenyl), 119.96, , 122.28, 125.05, , 128.70, 130.25; C=O, 156.69; QX(C3) phenyl, 122.05, 128.42, 129.47, 130.79, 131.84, 132.21, 133.41; QX(ring), 121.72, 121.86, 133.74, 137.98, 138.26, 139.50, 140.94.

Supplementary Table S1. Free-energy of binding (FEB) and interaction profile for n-butyl and isobutyl quinoxaline-7-carboxylate-1,4-di-*N*-oxide derivatives binding on the active site of *Tc*TR.

Compound	Free-energy of binding (FEB) kcal/mol	PLIP interaction profile		
		Hydrophobic	Hydrogen bond	Others
T-137	-6.8	Ile339(B), Asn340(B), Ile458(A)	Gly459(A), Glu466(A)	
T-138	-7.8	Pro336, Ile339, Ans340	Glu466(A)	
T-139	-6.9	Pro336(B), Ile339(B), Asn340(B), Phe396(A)	Glu466(A)	
T-140	-6.7	Pro336(B), Ile339(B), Asn340(B), Phe396(A)	Glu466(A)	
T-141	-7.9	Ile 339(B), Asn340(B), Phe396(A), Leu399(A), Ile458(A)	Gly459(A), Glu466(A)	
T-142	-8.1	Pro336(B), Ile339(B), Asn340(B), Phe396(A)		
T-143	-7.7	Ile339(B), Asn340(B), Phe396(A), Pro462(A)	His461(A), Glu466(A)	His461(A) (π-stacking)
T-144	-7.8	Pro336(B), Ile339(B), Asn340(B), Leu399(A), Ile458(A), Pro462(A), Glu466(A)	Glu466(A), Glu467(A)	His461(A) (π-stacking)
T-145	-7.5	Pro336(B), Ile339(B), Glu466(A)	Gly459(A)	His461(A) (salt bridge), Tyr455(A) (Halogen bond)
T-146	-7.3	Ile339(B), Asn340(B), Phe396(B)		

T-147	-7.3	Ile339(B), Asn340(B)	Gly459(A)	Pro336(B) (halogen bond), Arg472(A) (salt bridge)
T-148	-7.8	Pro336(B), Ile339(B), Asn340(B), Phe396(A)	Glu466(A)	
T-149	-8.3	Pro336(B), Ile339(B), Asn340(B)		
T-150	-9.9	Pro336(B), Ile339(B), Asn340(B), Phe396(A), Leu399(A), Pro462(A)		
T-151	-8.4	Pro336(B), Ile339(B), Phe396(A), Leu399(A), Pro462(A)	Glu466(A)	His461(A) (π-stacking)
T-155	-7	Pro336(B), Ile339(B), Glu466(A)	Gly459(A), Arg472(A)	His461(A) (salt bridge), Thr457(A) (halogen bond)
T-156	-7.9	Ile339(B), Asn340(B), Phe396(A), Ile458(A)		
T-157	-9	Pro336(B), Ile339(B), Phe396(A), Leu399(A), Pro462(A)		
T-158	-7.8	Pro336(B), Ile339(B), Asn340(B), Phe396(A)		
T-159	-7.4	Pro336(B), Ile339(B)	Glu466(A)	Arg472(A) (π-cation)
T-161	-7.5	Pro336(B), Ile339(B), Asn340(B), Phe396(A), Ile458(A), Glu466(A)		

T-162	-8.4	Ile339(B), Asn340(B), Phe396(A), Leu399(A), Ile458(A), Pro462(A)	Gly459(A), Glu466(A)	
T-163	-8.4	Pro336(B), Ile339(B), Asn340(B), Ile458(A), Pro462(A), Glu467(A)	Glu466(A), Glu467(A)	His461(A) (π-stacking)
T-164	-8.2	Ile339(B), Phe396(A), Glu466(A)	Gly459(A), Glu466(A)	
T-165	-8.1	Pro336(B), Ile339(B), Asn340(B), Leu399(A), Pro462(A), Glu466(A)	Glu466(A), Glu467(A)	His461(A) (π-stacking)
T-166	-7.1	Ile339(B), Asn340(B) Ile458(A), Glu466(A)	Gly459(A), Glu466(A)	
T-167	-8.4	Ile107(B), Pro336(B), Ile339(B), Asn340(B), Phe396(A), Leu399(A), Pro462(A)	Glu466(A)	His461(A) (π-stacking)
T-168	-7.8	Val54(B), Thr335(B), Ile339(B), Ala343(B)	Gly459(A)	His461(A) (salt bridge), Tyr455(A) (halogen bond)
T-169	-8.1	Ile339(B), Asn340(B), Phe396(A), Ile458(A), Pro462(A)	Gly459(A), His461(A), Glu466(A)	His461(A) (π-stacking)
T-170	-7	Ile339(B), Asn340(B), Ile458(A)	Gly459(A), Glu466(A)	

Trypanothione disulfide (1BZL)	-8.7	Trp22(B), Phe396(A)	Ser15(A), Glu19(B), Tyr111(B), Thr463(A), Glu466(A)	His461(A) (salt bridge)

Supplementary Table S2. Free-energy of binding (FEB) and 2D-interaction profile representation for all n-butyl and isobutyl quinoxaline-7-carboxylate-1,4-di-*N*-oxide derivatives binding on the active site of *Tc*TR.

2D-interaction profiles

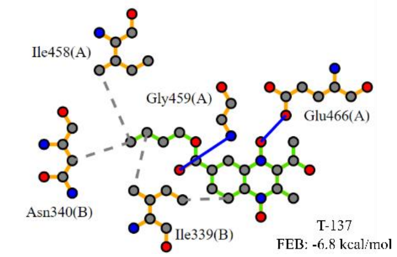

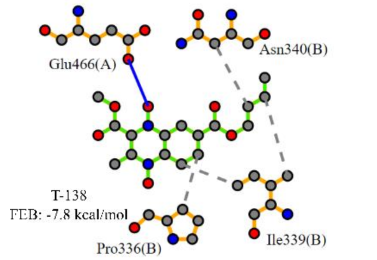

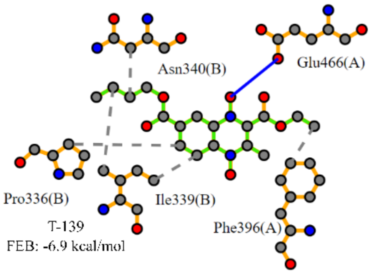

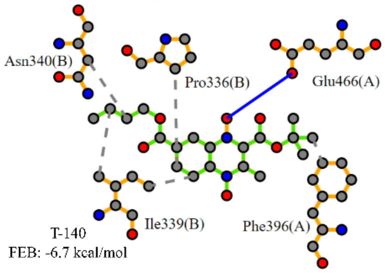

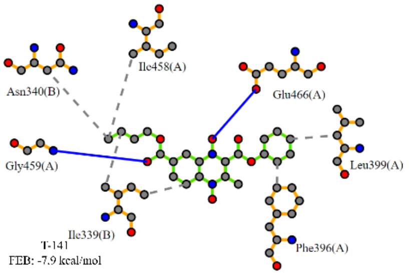

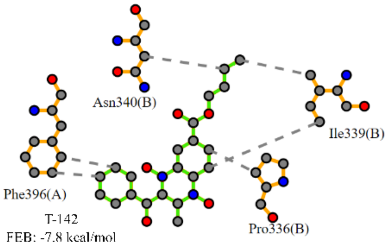

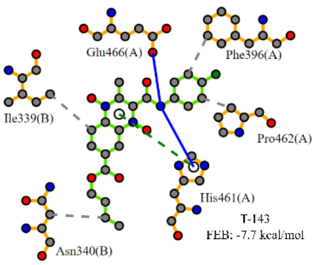

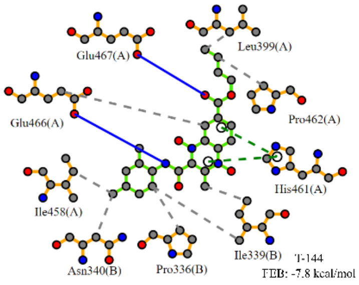

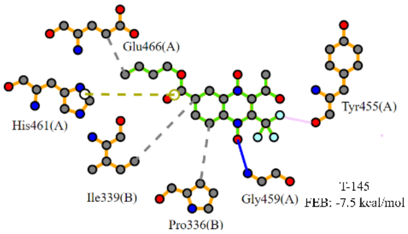

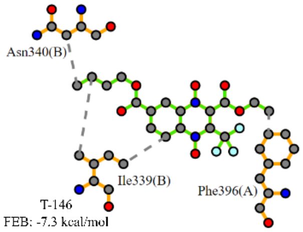

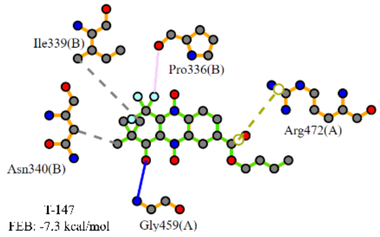

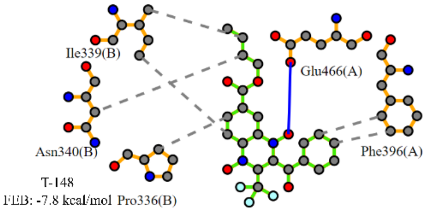

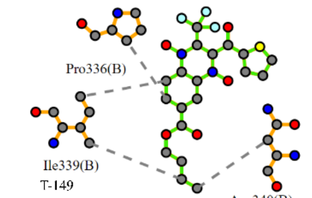

T-149
FEB: -8.3 kcal/mol

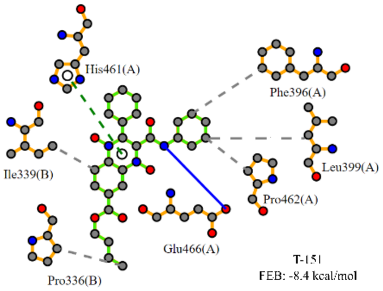

T-151
FEB: -8.4 kcal/mol

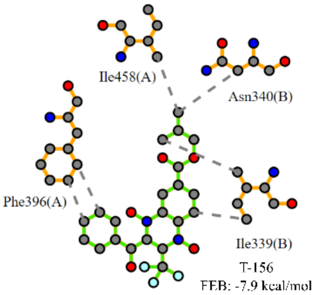

T-156
FEB: -7.9 kcal/mol

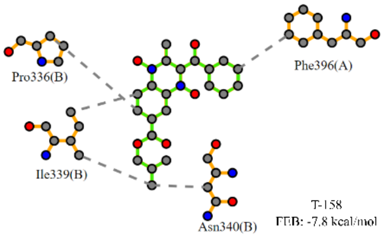

T-158
FEB: -7.8 kcal/mol

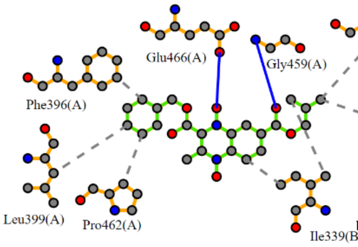

T-162
FEB: -8.4 kcal/mol

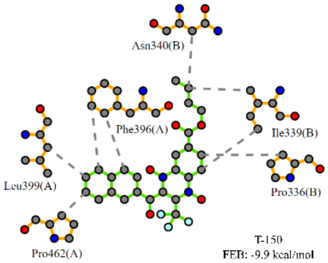

T-150
FEB: -9.9 kcal/mol

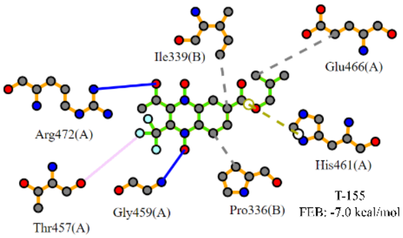

T-155
FEB: -7.0 kcal/mol

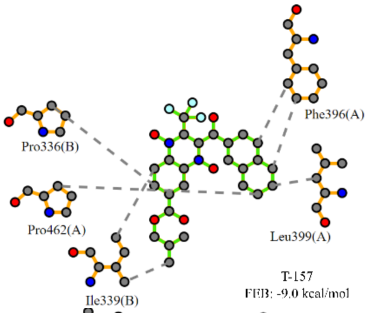

T-157
FEB: -9.0 kcal/mol

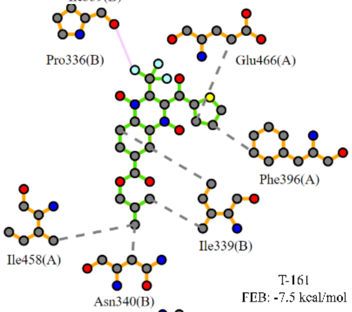

T-161
FEB: -7.5 kcal/mol

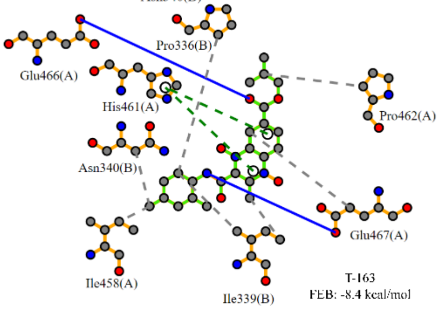

T-163
FEB: -8.4 kcal/mol

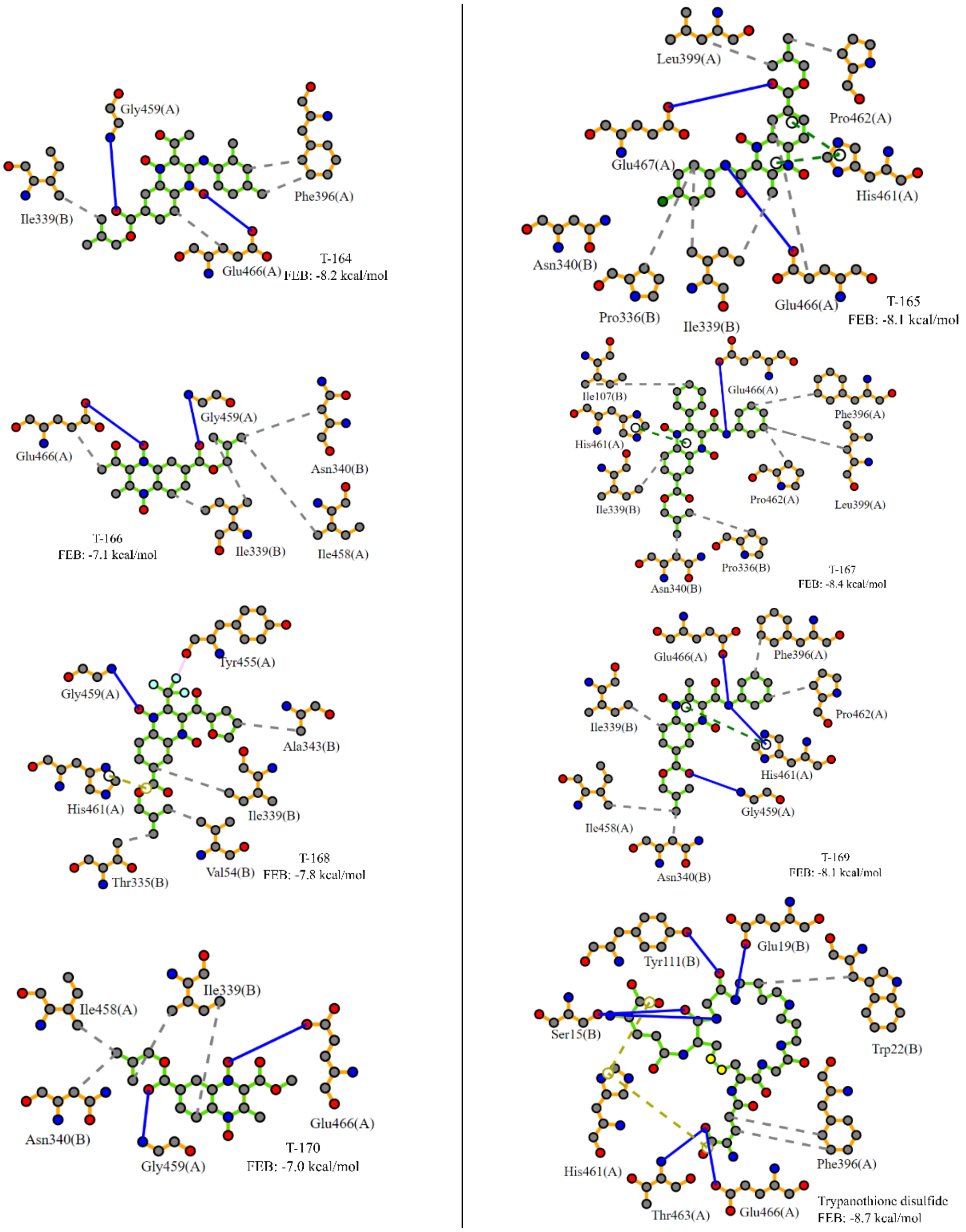

Interaction type legend: dashed grey line: hydrophobic interaction, solid blue line: hydrogen bond, dashed golden line: salt bridge, dashed green line: π-stacking, solid light violet line: halogen interaction.

3D-representation of T-147 and natural ligand trypanothione disulfide

Interaction profiles for top ranked docking poses for new TR inhibitor compound T-147 (a) and natural ligand trypanothione disulfide (b) with trypanothione reductase.

T-147

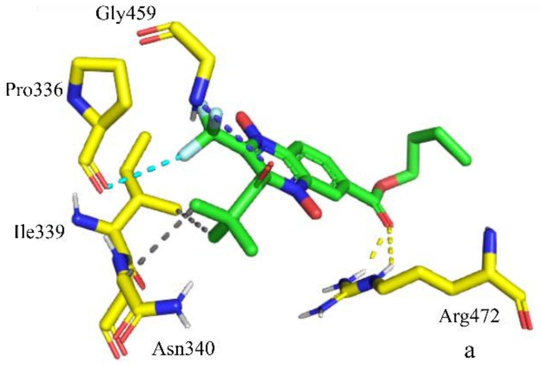

Trypanothione disulfide

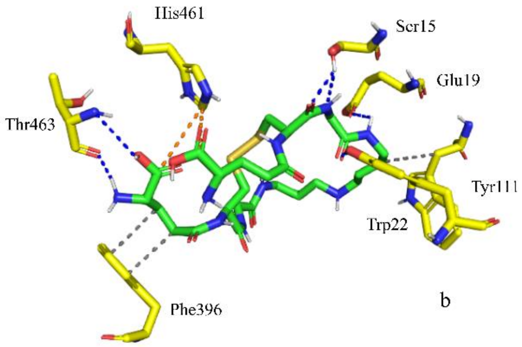

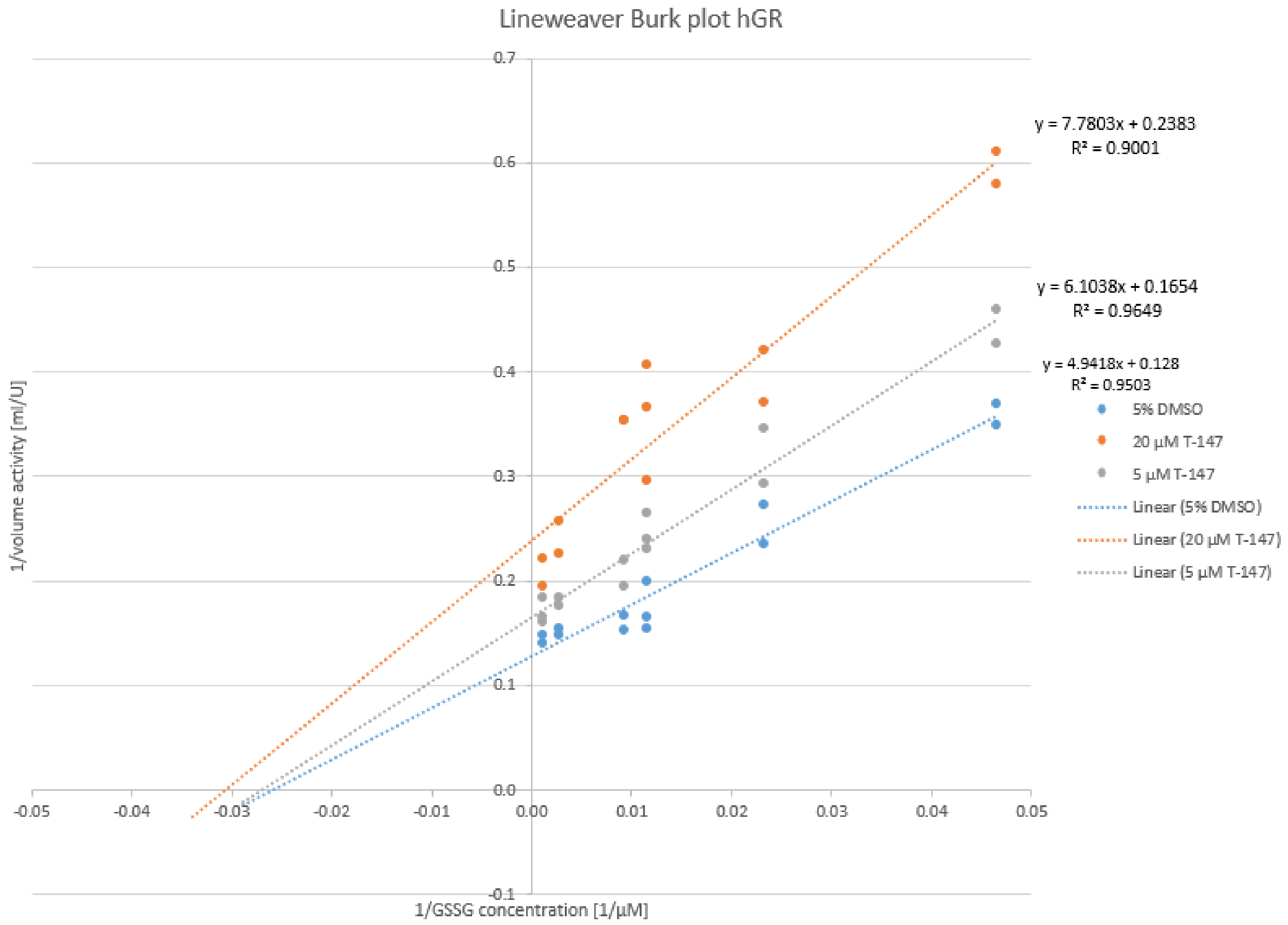

Supplementary Figure S1. Lineweaver Burk plot of GR inhibition by the compound T-147.

UPLC-MS

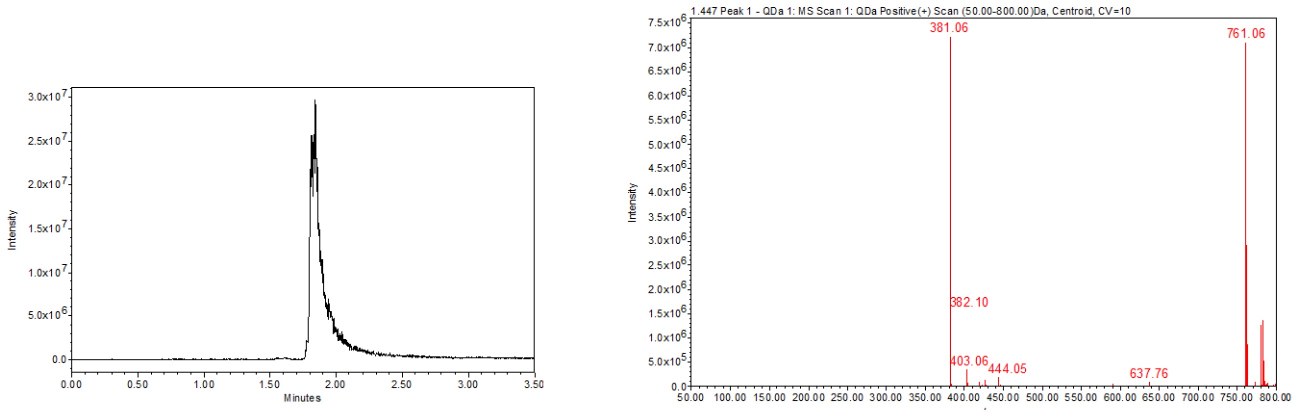

T-142 Chromatogram and mass spectrometry spectrum

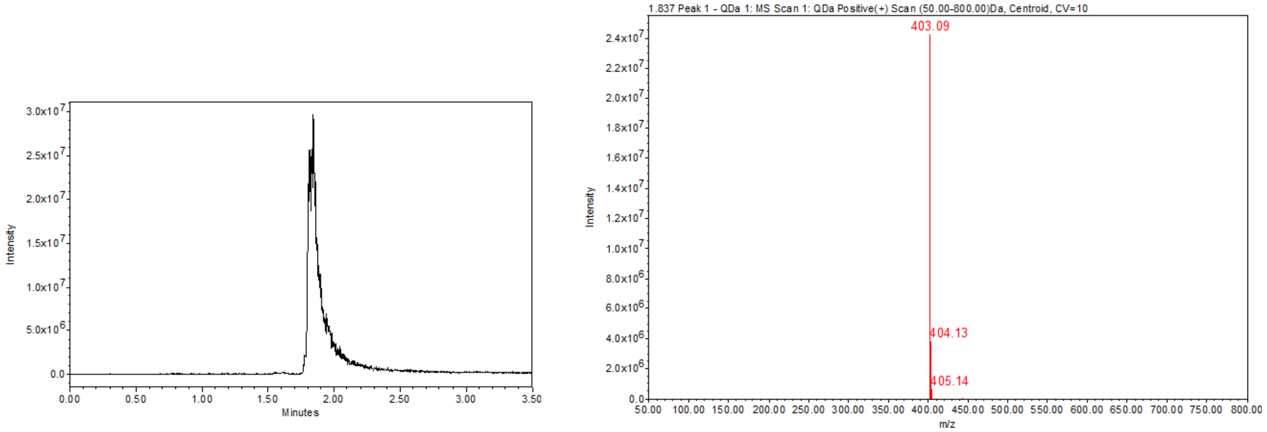

T-146 Chromatogram and mass spectrometry spectrum

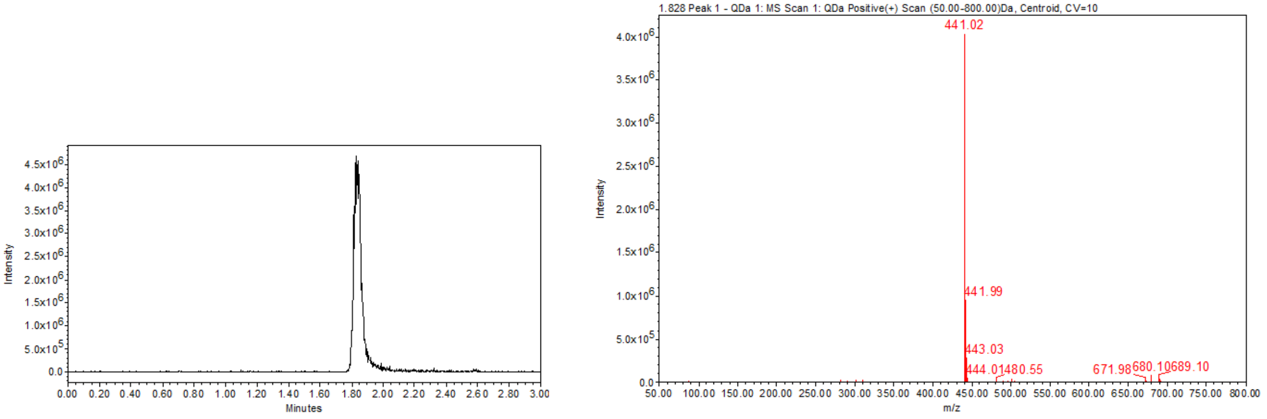

T-149 Chromatogram and mass spectrometry spectrum

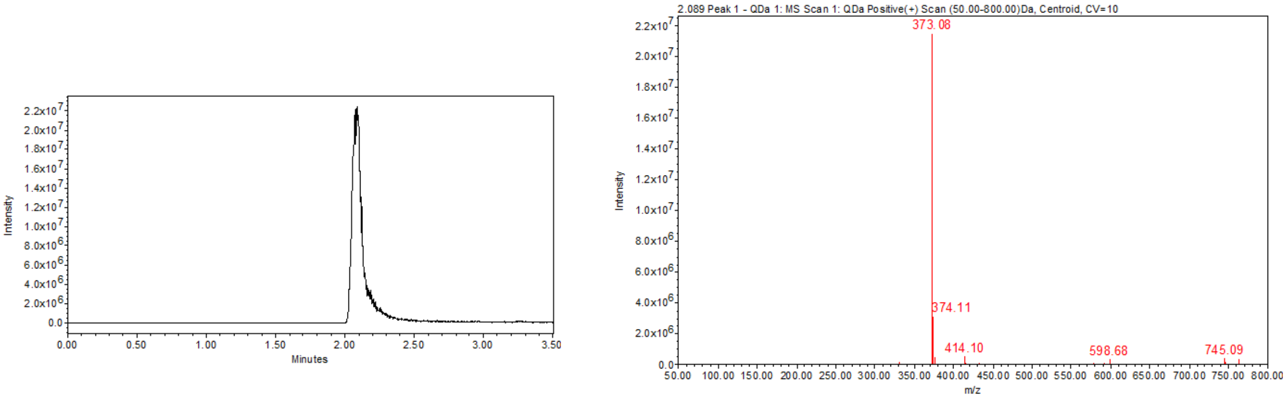

T-155 Chromatogram and mass spectrometry spectrum

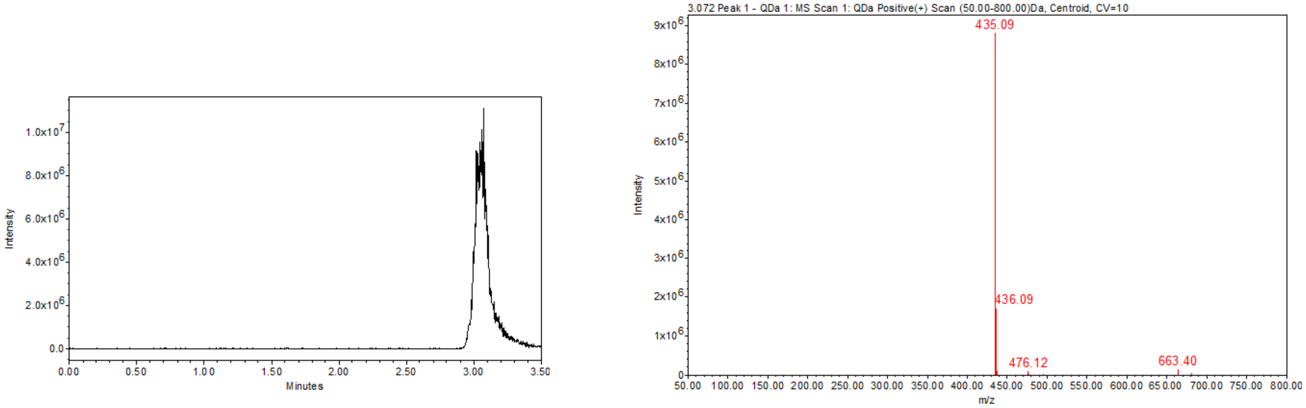

T-156 Chromatogram and mass spectrometry spectrum

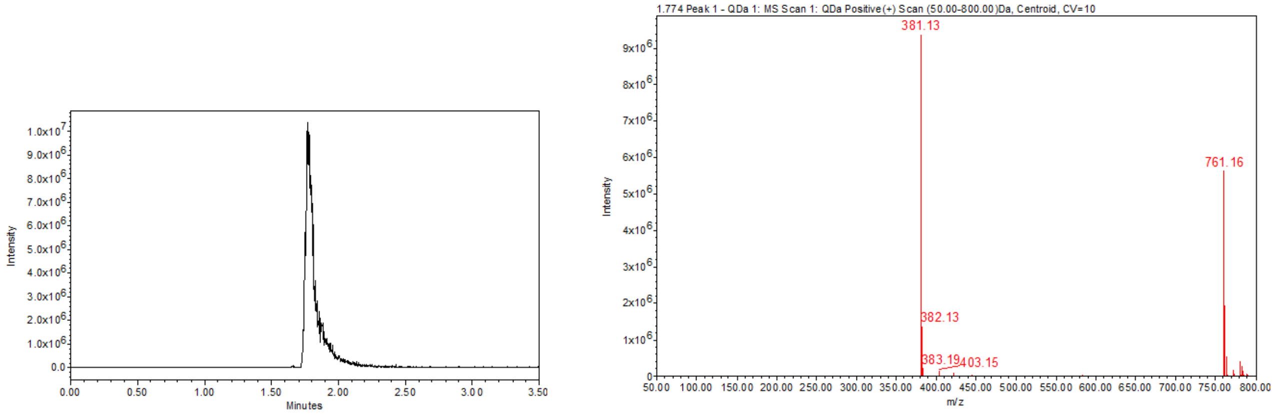

T-158 Chromatogram and mass spectrometry spectrum
